# Unmet Needs in Pharmacological Treatment of Apathy in Alzheimer’s Disease: A Systematic Review

**DOI:** 10.3389/fphar.2019.01108

**Published:** 2019-10-04

**Authors:** Christos G. Theleritis, Kostas T. Siarkos, Antonios M. Politis

**Affiliations:** ^1^Division of Geriatric Psychiatry, First Department of Psychiatry, National and Kapodistrian University of Athens, Athens, Greece; ^2^Department of Psychiatry and Behavioral Sciences, Johns Hopkins University, Baltimore, MD, United States

**Keywords:** apathy, pharmacological, treatment, dementia, Alzheimer’s disease

## Abstract

**Background:** Apathy is one of the most prevalent neuropsychiatric symptoms encountered in Alzheimer’s disease (AD) and may be an early sign in the development of dementia persisting over the disease course. It has been associated with poor disease outcome, impaired daily functioning, and significant caregiver distress. Early diagnosis and timely treatment of apathy in AD are of great importance. However, approved agents for apathy are still missing.

**Methods:** Within this context, we conducted an extensive electronic search in the databases included in the National Library of Medicine, PsychInfo, and Google Scholar for studies that have investigated the effect of pharmacological treatments in apathy in AD. There were no limitations regarding study design and all care settings were considered for inclusion. Structured measures for level of evidence and study quality were employed to evaluate the results.

**Results:** A total of 1,607 records were identified; 1,483 records remained after the removal of duplicates and were screened; 166 full-text articles were selected and assessed for eligibility and a remaining 90 unique studies and relevant reviews were included in the qualitative synthesis. Acetylcholinesterase inhibitors, gingko biloba, and methylphenidate were found to be successful in reducing apathy in patients with AD. Methodological heterogeneity in the studies and the small amount of studies where apathy was the primary outcome are limiting factors to assess for group effects.

**Conclusions:** Pharmacological treatment of apathy in AD is an underexplored field. Standardized and systematic efforts are needed to establish a possible treatment benefit. Elucidating the pathophysiology of apathy and its components or subtypes will inform disease models and mechanistic drug studies that can quantify a benefit from specific agents for specific AD groups.

## Introduction

Applying the neuropsychiatric inventory (NPI), Mega et al. (1996) found that 88% of patients with Alzheimer’s disease (AD) had neuropsychiatric symptoms, of which apathy was the most frequent, reported to occur in 27% to 72% of patients (Cummings, 1997; Benoit et al., 1999; Lyketsos et al., 2000; Lyketsos et al., 2002; Lyketsos et al., 2011). Apathy has been defined as the absence or lack of feeling, emotion, interest, concern, or motivation not attributable to a decreased level of consciousness, cognitive impairment, or emotional distress (Marin, 1990). Starkstein et al. (2001) proposed the following core features of apathy: diminished motivation, diminished initiative and interest, and blunting of emotions. Recently, proposed diagnostic criteria (Robert et al., 2018) define “apathy” as a quantitative reduction of goal-directed activity that persists for at least 4 weeks, affects at least two of the three apathy dimensions (behavior/cognition, emotion, social interaction), is not fully explained by effects of a substance or major changes in the patient’s environment, and is accompanied by identifiable functional impairments (Mulin et al., 2011). In patients with apathy, the capacity of the frontal cortex to select, initiate, maintain, and shift programs of action is undermined (Levy and Dubois, 2006). In dementia, Lyketsos et al. (2004) proposed that apathy is an aspect of executive dysfunction syndrome and is probably caused by damage to frontal–subcortical brain circuits. Indeed, apathy is correlated with neuronal loss, higher tangle counts, white matter hyperintensities, and hypoperfusion in regions involved in frontal–subcortical networks (Theleritis et al., 2014; Le Heron et al., 2018). Apathy frequently complicates the course and management of dementia and is prevalent in patients even with milder forms of cognitive impairment in clinic- (Drijgers et al., 2011) and community-based (Lyketsos et al., 2002; Clarke et al., 2010) samples. Onyike et al. (2007) proposed that apathy is an early sign of cognitive decline. Consequently, apathy has been associated with reduced daily functioning, functional disability, self-neglect, behaviors evoking embarrassment, caregiver distress, and poor outcome (Landes et al., 2001; Politis et al., 2004). Within this context, early diagnosis and effective treatment of apathetic patients with AD are of great importance. Although apathy is a prevalent neuropsychiatric syndrome, no specific treatment for apathy in AD has been approved. Clinical apathy implying motor, cognitive, affective, and behavioral symptoms suggests a benefit may systematically arise from different or close related or broad treatment classes and interestingly when combinations among them are tested.

## Methods

Inclusion and exclusion criteria: We searched for studies on pharmacological treatment of apathy in AD. We unrestrictedly included group studies, with patients diagnosed with AD using clinical criteria and structured tools treated with pharmacological agents in controlled and uncontrolled designs and an outcome measure on apathy is reported. Other neuropsychiatric manifestations and concomitant psychoactive medications were allowed. Other neurological conditions and dementias other than AD, drug abuse, and severe systematic or malignant conditions were exclusion criteria. Relevant reviews were also considered for inclusion. Reports on single cases were excluded.

Search strategy and study selection: The most current search was conducted on March 31, 2019. Building upon our previous review (Theleritis et al., 2017) we adopted the same search method in order to identify pharmacological studies relevant to the treatment of apathy in AD, from an extensive electronic search from the databases included in the National Library of Medicine for “apathy and dementia,” as well as PsychInfo and Google Scholar. Further articles for inclusion were identified by searching the references of retrieved articles and by checking the Cochrane library. The following search terms were also used: apathy, abulia, amotivation, or passivity, dementia, Alzheimer* disease, treatment, management, pharmacological, drug, donepezil, galantamine, rivastigmine, memantine, atypical antipsychotic, risperidone, olanzapine, amisulpride, antidepressants, conventional antipsychotics, stimulant, psychostimulant, methylphenidate, modafinil, anticonvulsants, and antiparkinsonic drugs. Articles that involved patients with dementia other than AD or that did not report a specific outcome measure of apathy were excluded. Three authors have gone through all the abstracts; when there was disagreement between the three authors, the issue was resolved by a consensus meeting with the last author. For pharmacological treatment of apathy, there were no limitations regarding study design, and thus, the review included meta-analyses, randomized controlled trials (RCTs), and open-label studies. All selected articles were read in full, and their level of evidence and outcome were assessed by all the authors. All care settings were considered for inclusion. We did not search for unpublished studies. The studies retained for inclusion were classified by their level of evidence following the system of the Medicine OCfE-b (2009). Grades of recommendation were also scored with this classification (**Table 1**). All RCTs were further evaluated with the use of the PEDro rating scale (Maher et al., 2003). It comprises 11 items as follows: participant eligibility criteria and source specified, random allocation of participants to interventions, allocation concealed, intervention groups similar at baseline regarding key outcome measures and important prognostic indicators, blinded subjects, blinded therapists who administered the intervention, blinded assessors who measured at least 1 key outcome, dropouts (attrition bias), intention to treat analysis, reported between group statistical comparisons, and reported measures of variability. Each item was evaluated (items 2–11) and added to give a total score. Trials were then qualitatively described according to PEDro scores as follows: a score of 7 or greater was “high” quality, a score of 5 or 6 was “moderate” quality, and a score of 4 or less was “poor” quality (Harvey et al., 2002).

**Table 1:** Levels of evidence and grades of recommendation of OCEBM^a^.

Level Explanation
Levels of evidence
1. One or more RCTs (or systematic review of trials) of sufficient size to ensure a low risk of false-positive or false-negative results (narrow confidence interval).
2. Good quality cohort studies or low-quality RCT (eg, too small, <80% follow-up).
3. Case–control studies, including systematic reviews of case–control studies.
4. Case series and poor quality cohort and case–control studies.
5. Expert opinion without explicit critical appraisal or based on physiology, bench research, or ‘‘first principles.’’
Grades/strengths of recommendations
A. Consistent level 1 studies.
B. Consistent level 2 or 3 studies or extrapolations from level 1 studies.
C. Level 4 studies or extrapolations from level 2 or 3 studies.
D. Level 5 evidence or troublingly inconsistent or inconclusive studies of any label.

OCEBM, Oxford Centre for Evidence-Based Medicine; RCTs, randomized controlled trials.

a Summarized from Medicine OCfE-b, (2009).

## Results—Review of Pharmacological Treatments

**Figure 1:**
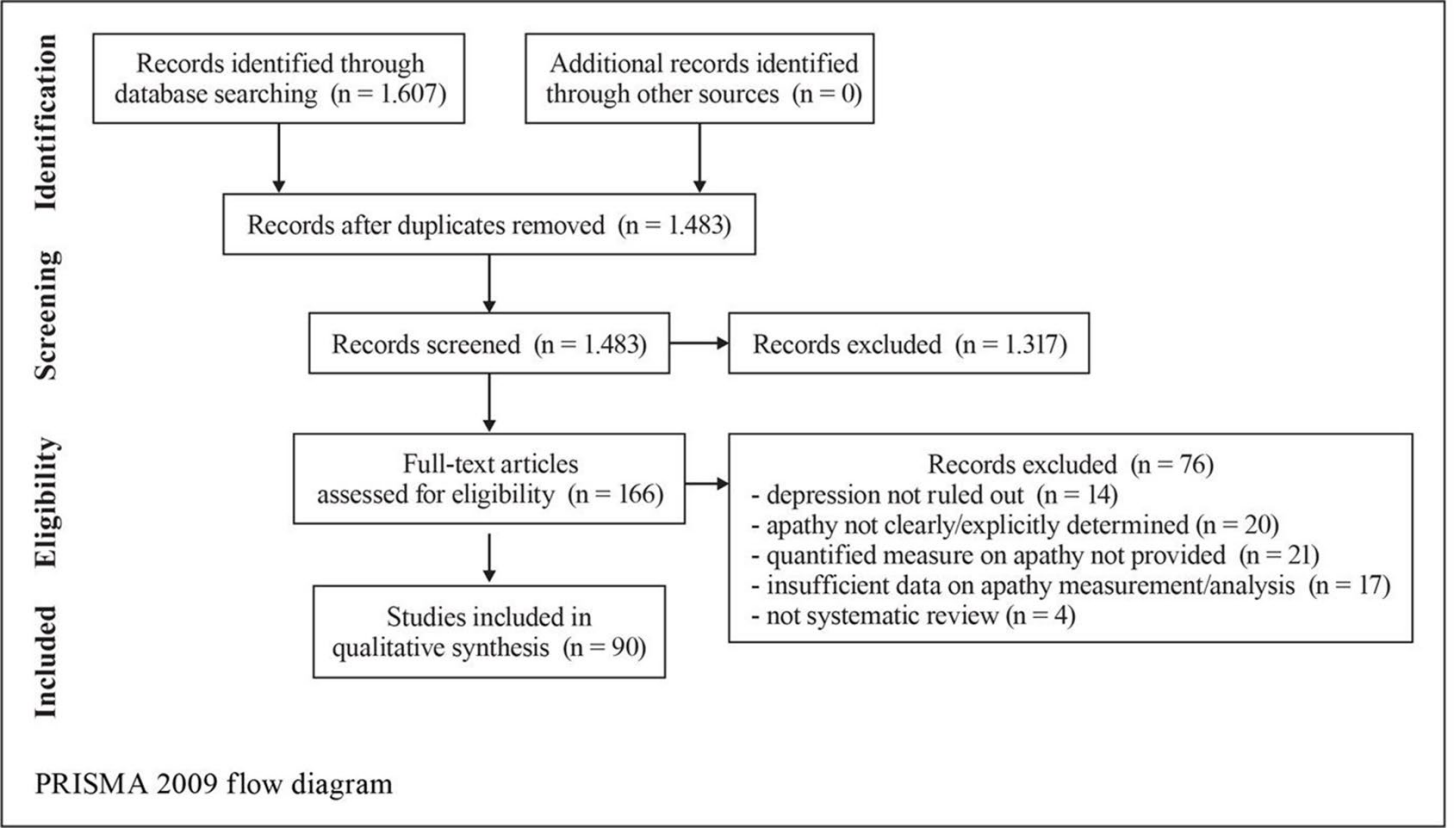
PRISMA 2009 flow diagram.

A search using the keywords “apathy” and “dementia” yielded 1,607 results (**Figure 1**). When AD was chosen instead of dementia, results were limited to 877. The combination of keywords “apathy” AND “Alzheimer* disease” AND “treatment” yielded 420 results; of apathy AND Alzheimer* disease AND “pharmacological treatment” 166 results. In the final review, 60 pharmacological studies, 4 pooled data analyses on donepezil (N = 490), galantamine (N = 2033), mematine (N = 2311), and metrifonate (N = 672), respectively, and two meta-analyses (N = 6384 and N = 4867, respectively) were considered. The majority of the studies were of high quality according to PEDro scores and relatively high level of OCEBM evidence (**Table 3**). However, effect sizes were small and multiple heterogeneity exists. Description of the results is following (see also **Table 1**).

### Donepezil

Sixteen studies (seven RCTs and nine open-label studies) were found to assess the efficacy of donepezil in the treatment of apathy in AD (**Tables 2** and **3**). Apathy was assessed in 12 studies using the NPI, in one with the Top Symptoms (TOPS) checklist and the NPI, in one with consortium to establish a registry for Alzheimer’s disease (CERAD) Behavior Rating Scale for Dementia (BRSD), and in two with the Apathy Evaluation Scale (AES). All studies except two demonstrated at least some benefit in apathy scores after treatment with donepezil.

**Table 2:** Randomized, Placebo-Controlled Trials of Pharmacological agents for apathy in Alzheimer’s disease.

Trial	N (ADG/C)	Intervention	Treatment duration (weeks)	Apathy scale (primary)	Outcome*	Comments
**Donepezil**						
Tariot et al. (2001)	208 (103/105)	Donepezil (up to 10 mg/day)	24	NPI (NPI)	NEGATIVE	- Long term care setting. AD with CVD included. - Similar rates and severities of AEs except of weight loss, abdominal pain, nausea, tremor, peripheral edema, myasthenia, back pain, stupor (twice the frequency in ≥ 5% of the AD group receiving donepezil).
Feldman et al. (2001) (Donepezil MSAD Study Investigators Group)	290 (144/146)	Donepezil (up to 10 mg/day)	24	NPI (CIBIC+)	POSITIVE only in the psychoactive-free patient subgroup	- sMMSE: 5-18. - Differences reported for NPI total scores. - Similar overall AEs except diarrhea, headache, arthralgia, vomiting (twice the frequency in ≥ 5% of the AD group receiving donepezil).
Gauthier et al. (2002)	290 (144/146)	Donepezil (up to 10 mg/day)	24	NPI (CIBIC+)	POSITIVE	- Sub analysis of Feldman et al. (2001). - Differences reported for NPI total scores. - Not significantly preventive in symptom-free patients at baseline.
Feldman et al. (2005)	290 (144/146)	Donepezil (up to 10 mg/day)	24	NPI (CIBIC+)	POSITIVE	- Subgroup analysis of the Feldman et al. (2001) study. - sMMSE: 5-12.
Holmes et al. (2004)	96 (41/55)	Donepezil (10 mg/day)	12	NPI (NPI)	POSITIVE NPI-total (NEGATIVE in the Observed Case Analysis)	- 12-week open label followed by a 12-week RCT. - Mean MMSE at randomization: 20.8 AD vs 21.1 NC. - Safety rates not reported.
Seltzer et al. (2004)	153 (96/57)	Donepezil (up to 10 mg/day)	24	AES (mADAS-cog)	NEGATIVE	- Mean MMSE: 24.2.- Similar safety rates between groups.
Cummings et al. (2006a)	120	Donepezil (10 mg/day)	20	NPI (NPI)	POSITIVE	- Post hoc analysis on the Donepezil+ placebo data from a 12-week RCT on Donepezil + Sertraline or Placebo and 8-weeks open label administration. - Relatively severe psychopathology (NPI: 30.5) in drug-free patients at baseline. - Safety outcomes not reported.
Winblad et al. (2001)(Donepezil Nordic Study Group)	286 (142/144)	Donepezil (up to 10 mg/day)	52 (1 year)	NPI (Gottfries-Bråne-Steen scale)	NEGATIVE	- MMSE: 10-26 - At least one serious adverse event in the Donepezil vs Placebo group: 25 % - 14 %. vertigo, asthenia, syncope (twice the frequency in ≥ 5% of the AD group receiving donepezil) - Relatively high dropout rates
**Galantamine**						
Tariot et al. (2000)	978 (692/286)	Up to 24 mg/day	20	NPI (ADAS-cog, CIBIC+)	POSITIVE	-Mean MMSE: 18. - Significant effects with 24mg. - Similar safety rates between groups. - Small dose-related weight loss effect in the galantamine group.
Rockwood et al. (2001)	386 (261/125)	Up to 32 mg/day	12	NPI (ADAS-cog, CIBIC+)	NEGATIVE	- Mean MMSE: 19.7. - At least 13-fold higher discontinuation rates due to AEs in the treatment group. Nausea, dizziness, vomiting, anorexia, somnolence, abdominal pain, agitation occurred in ≥ 5% more often with galantamine than with placebo (serious AEs in 8 % and 6 % respectively).
Erkinjutti et al. (2002)	592 (396/196)	24 mg/day	24	NPI (ADAS-cog, CIBIC+)	POSITIVE (not enough powered to detect individual NPI items differences)	- MMSE: 10-25. - VaD patients also included (N = 188). - Discontinuation rates due to AEs were 19.7 and 8.2 for patients and controls respectively.
Cummings et al. (2004)	978 (692/286)	Up to 24 mg/day	21	NPI (NPI)	POSITIVE (NEGATIVE for apathy)	- Data analysis from Tariot et al. (2000). - Relatively low NPI baseline scores. - No adjustment for ADAS-cog and CIBIC+ scores for drug-placebo differences as these scales were not the primary outcome measures in this analysis. - Significant drug-placebo differences revealed for 16 and 24 mg/day. - Significantly less new apathy symptoms in psychopathology -free patients at baseline assigned to galantamine.
**Memantine**						
Pantev et al. (1993)	60	Up to 30 mg/day	4	Sandoz Clinical Assessment-Geriatric scale (SCAG), NOSIE	POSITIVE	
Winblad and Poritis (1999)	166 (82/84) 151 treated per protocol	10 mg/day	12	CGI-C, (Behavioral Rating Scale for Geriatric Patients - BGP)	POSITIVE	- Mean MMSE: 6.3. - 49 % AD 51 % VaD. - Similar rates of AEs and death reported. In all AEs cases a causal relationship to the trial medication was rated as ‘unlikely’ by the investigators. - Non-specific apathy measure.
Cummings et al. (2006b)	404 (201/203) (200/200)	20mg/day	24	12-item NPI (SIB, modified ADCS-ADL)	NEGATIVE	- Mean MMSE: 10. - Post-hoc exploratory analysis of a secondary outcome. - Concomitant Donepezil. - Treatment discontinuation for memantine vs placebo were 15 (7.4 %) vs 25 (12.4 %), respectively. Confusion and headache occurred in ≥ 5, in the memantine group and at least twice as much than in the placebo group.
**Ginkgo Biloba**						
Scripnikov et al. (2007)	400	240mg	22	NPI (SKT)	POSITIVE	- SKT 9-23 and NPI ≥ 5. - Possible AD with CVD and VaD also included. - Safe and well tolerated, with lower numbers of adverse events and serious adverse events in the active treatment group vs. placebo group (specifically, headache and dizziness).
Bachinskaya et al. (2011)	404 (202/202)	240 mg	24	NPI (NPI)	POSITIVE	- Mild-to-moderate dementia. NPI ≥ 5 and at least one item ≥ 3. AD with or without CVD, VD also included.
Ihl et al. (2011)	410 (206/204)	240 mg	24	NPI (NPI)	POSITIVE	- Mild-to-moderate dementia. NPI ≥ 5 and at least one item ≥ 3. AD with or without CVD, VD also included. Safe and well tolerated, with a lower number of adverse events in the active treatment group vs. placebo group (specifically, dizziness and tinnitus).
**Methylphenidate**						
Hermann et al. (2008)	13	10mg/day	5	AES (AES)	POSITIVE	- All participants were stabilized on an AChEI for at least 3 months. - A significantly greater proportion of patients had ≥ 1 AE with methylphenidate compared with placebo (3 vs 1; χ2 = 4.33, P = 0.038) including delusions, agitation, anger, irritability, and insomnia, which resolved upon drug discontinuation. - Dose reduced in one patient due to irregular heartbeat.
Rosenberg et al. (2013)	60 (29/31)	20mg/day	6	AES/NPI (AES)	NEGATIVE (AES) POSITIVE (total NPI)	- Trends toward significance for greater anxiety [OR = 2.7, 95% CI(0.9, 7.8) P = .07], weight loss > 2% [OR = 3.7, 95%CI(0.9, 19.4) P = .06] in the methylphenidate-treated group, and more frequent arthralgia [OR = 0.3, 95%CI(0.1, 0.9; P = 0.03] in the placebo participants. - Four methylphenidate participants and two placebo participants discontinued due to hypertension, nervousness, nausea, anxiety, and insomnia, drop in hemoglobin respectively.
Padala et al. (2017)	60 (30/30)	9.5mg/day	12	AES-C, 3MS, MMSE, CGI-I, CGI-S, (AES-C)	POSITIVE	- Study in males. - Treatment effect over time was independent from baseline depression presence, severity as well as from antidepressant medication and AchEIs. - Higher systolic blood pressure observed in the methylphenidate group (median increase of 7mmHg, p < 0.001).
**Modafinil**						
Frakey et al. (2012)	22 (11/11)	Up to 200mg/day	8	Frontal Systems Behavior Scale	NEGATIVE	- Concomitant stable doses of an AchEI. - No safety reports.
**Citalopram**						
Porsteinsson et al. (2014)	186 (94/92)	Up to 30mg/day	9	NPI (NBRS-A, mADCS-CGI-C)	POSITIVE (total NPI score)	- Citalopram argued for cognitive dysfunction.

Displayed are the RCTs reviewed after excluding the overlapping studies. *Outcome rated for statistically significant results at p < 0.05 favoring the specific treatment for apathy (positive) or not (negative). Abbreviations: AChEI(s) : acetylcholinesterase inhibitor(s); ADG/C: Active Drug Group/ Controls; IG/C: Intervention Group/Controls; (Primary): Primary Outcome measure for the study; AD: Alzheimer’s Disease; VaD: Vascular dementia; AEs: adverse events; CVD: cerebrovascular disease; MMSE: Mini Mental State Examination; SIB: Severe Impairment Battery; ADCS-ADL: AD Cooperative Study-Activites of Daily Living inventory; DAIR: Dementia Apathy Interview and Rating; SKT: Short Cognitive Performance test; mADCS-CGI-C: modified Alzheimer Disease Cooperative Study-Clinical Global Impression of Change inventory; NBRS-A: Neurobehavioral Rating Scale; AES: Apathy Evaluation Scale; 3MS: Modified Mini Mental Examination; CGI-I: Clinical Global Impression scale-Improvement; CGI-S: Clinical Global Impression scale-Severity; AI: Apathy Inventory; MSS: Multi-sensory stimulation; BRS: Behavioral Rating Scale; BMD: Behavior and Mood Disturbance Scale, DCM: Dementia Care Mapping; CIBIC+:Clinician’s Interview-Based Impression of Change with caregiver input; SCAG: Sandoz Clinical Assessment-Geriatric Scale; MOSES: Multidimensional Observation Scale for Elderly Subjects; NOSIE: Nurses Observation Scale for Inpatient Evaluation.

**Table 3:** Quality rating for the pharmacological/biological studies reviewed.

Pharmacological/ Biological therapies	PEDro	PEDro	PEDro	PEDro	PEDro	PEDro	PEDro	PEDro	PEDro	PEDro	PEDro	PEDro	OCEBM
	Random group allocation	Allocation concealed	Baseline group similarity	Blinding of all subjects	Blinding of all therapists	Blinding of all assessors of at least one key outcome	Less than 15% dropouts	Intention to treat analysis of at least one key outcome	Between group statistical comparisons reported for at least one key outcome	Point measurements and measurements of variability (range, interquartile range, variance, and SD) provided for at least one key outcome	Total ‘yes’ Score	Quality rate	OCEBM
**Donepezil**													
Cummings et al., 2006a (Donepezil+Sertraline)	Y	N	Y	Y	Y	Y	N	Y	Y	Y	8	high	A
Feldman et al., 2001	Y	Y	Y	Y	Y	Y	N	Y	Y	Y	9	high	A
Tariot et al., 2001	Y	Y	Y	Y	Y	Y	N	Y	Y	Y	9	high	A
Holmes et al., 2004	Y	Y	Y	Y	Y	Y	N	Y	Y	Y	9	high	A
Seltzer et al., 2004	Y	N	N	Y	Y	Y	N	Y	N	N	5	moderate	A
Gauthier et al., 2002	Y	Y	Y	Y	Y	Y	N	Y	Y	Y	9	high	A
Feldman et al., 2005	Y	Y	Y	Y	Y	Y	N	Y	Y	Y	9	high	A
Winblad et al., 2001	Y	N	Y	Y	Y	Y	N	Y	N	N	6	moderate	B
Rea et al., 2015 (Donepezil+choline alphoscerate)	Y	Y	Y	Y	Y	Y	N	N	Y	Y	8	high	B
**Galantamine**													
Tariot et al., 2000	Y	Y	Y	Y	Y	Y	N	Y	Y	Y	9	high	A
Rockwood et al., 2001	Y	Y	Y	Y	Y	Y	N	Y	Y	Y	9	high	A
Erkinjuntti et al., 2002	Y	Y	Y	Y	Y	Y	N	Y	Y	Y	9	high	A
Cummings et al., 2004	Y	Y	Y	Y	Y	Y	N	Y	Y	Y	9	high	A
**Memantine**													
Winblad and Poritis, 1999	Y	N	Y	Y	Y	Y	Y	Y	Y	Y	9	high	A
Cummings et al., 2006b	Y	Y	Y	Y	Y	Y	N	Y	Y	Y	9	high	A
Zhou et al., 2019 (Memantine+Citalopram)	Y	N	Y	Y	N	N	Y	N	Y	Y	6	moderate	B
**Methylphenidate**													
Hermann et al., 2008	Y	N	N	Y	Y	Y	N	N	Y	Y	6	moderate	A
Rosenberg et al., 2013	Y	Y	Y	Y	Y	Y	Y	Y	Y	Y	10	high	A
Padala et al., 2017	Y	Y	Y*	Y	Y	Y	Y	Y	Y	Y	10	high	A
**Modafinil**													
Frakey et al., 2012	Y	N	Y	Y	Y	Y	Y	N	Y	Y	8	high	A
Ginkgo Biloba													
Scripnikov et al., 2007	N	N	Y	Y	Y	Y	N	N	Y	Y	7	high	A
Bachinskaya et al., 2011	Y	N	Y	Y	Y	Y	N	Y	Y	Y	8	high	A
Ihl et al., 2011	Y	N	Y	Y	Y	Y	Y	Y	Y	Y	9	high	A
**Tacrine**													
Ahlin et al., 1991	Y	N	Y	Y	Y	Y	Y	N	Y	Y	8	high	A
**Metrifonate**													
Kaufer, 1998	Y	N	Y	Y	Y	Y	N	Y	Y	N	7	high	A
Dubois et al., 1999	Y	N	Y	Y	Y	Y	Y	Y	Y	Y	9	high	A

PEDro, PEDro Rating Scale; OCEBM, Levels of Evidence and Grades of Recommendation with Oxford Centre for Evidence-Based Medicine System. *Although groups differed significantly in terms of apathy severity (p = 0.024), activities of daily living (p = 0.006, p = 0.033) and Clinical Global Impression severity (p = 0.011) at baseline this is considered arising by chance with random allocation.

*Randomized controlled trials*: Tariot et al. (2001) in a 24-week RCT with 208 patients, did not find a significant difference in apathy NPI scores between donepezil-treated and placebo groups. Feldman et al. (2001) conducted a 24-week RCT in 290 patients. A retrospective sub-analysis (Gauthier et al., 2002) of individuals’ NPI items from this study (Feldman et al., 2001) has shown significant differences in NPI scores for apathy, following treatment with donepezil versus placebo. A subsequent sub-analysis (Feldman et al., 2005) of the same RCT (Feldman et al., 2001) in 145 patients with severe AD also found a significant improvement in NPI apathy score with donepezil. In the Clinician’s Interview-Based Impression of Change with caregiver input (CIBICþ) scale, patient and caregiver input on clinical, mental/cognitive, behavior, and functioning areas was received and rated on a seven-category scale (three categories for worsening, one for no change, and three for improvement) to make a composite change rater estimation. In a study by Holmes et al. (2004), 134 patients were treated openly with donepezil for 12 weeks, then they were randomized (60:40) to either placebo or 10 mg donepezil daily for another 12 weeks. Significant improvement in NPI apathy scores was observed after treatment. In a 24-week multicenter RCT by Seltzer et al. (2004), the efficacy of donepezil was assessed in 153 patients. On the AS, the donepezil-treated group tended to score higher versus placebo, but no significant difference was detected, probably because patients had only mild apathy at baseline. Cummings et al. (2006a) have conducted a secondary analysis of a three-phase study involving donepezil and sertraline. Factor analysis of the baseline NPI-12 data revealed that a factor that comprised depression, anxiety, and apathy had a 27% reduction from baseline to final assessment (effect size = 0.39) in the 120 patients treated with donepezil. In an RCT by Rea et al. (2015) in patients with AD, the efficacy of cholinergic precursor choline alphoscerate and cholinesterase inhibitor donepezil versus donepezil alone on symptoms of apathy was investigated. Apathy severity was positively related to frontal assessment battery (FAB) scores (correlation: 0.404, P -.001). In these patients, namely those with normal FAB scores, the difference between the two treatments was statistically significant, while it was not significant in patients having a very low and mild FAB scores. Data were collected retrospectively, sample size was limited, and apathy was measured with NPI and was a secondary outcome of the association between the cholinesterase inhibitor donepezil and the cholinergic precursor choline alphoscerate in AD (ASCOMALVA).

*Open-label studies*: In an open-label retrospective study by Mega et al. (1999), behavioral improvement was seen in 35 patients, worsening in 24, and no change in 27 of the 86 participants treated with donepezil. Apathy NPI scores significantly improved for responders versus nonresponders. In an 18-month open-label study by Matthews et al. (2000), 80 patients participated. Apathy symptoms (assessed with NPI) reduced or cleared in 93% of patients treated with donepezil. In a 12-month open-label study by Weiner et al. (2000), 25 patients participated. Improvement in apathy symptoms (assessed with The CERAD BRSD) was found after treatment with donepezil. Barak et al. (2001) conducted a 24-week open-label study assessing the efficacy of donepezil in 10 patients. Apathy/indifference NPI score was reduced after 6-month treatment. In a 6-month open-label study by Paleacu et al. (2002), 28 patients participated. Mean apathy NPI score was improved after 6-month treatment. Tanaka et al. (2004), in a 12-week open-label study, assessed the efficacy of donepezil in 70 patients. After treatment, 21 patients showed a behavioral response, 42 showed no behavioral change, and 7 worsened. Apathy NPI score significantly improved among the responder group. Rockwood et al. (2007) in a multicenter, 6-month, open-label study, investigated the efficacy of donepezil in 101 patients. Apathy was assessed with the TOPS checklist and the NPI and was one of the two symptoms that benefited the most from treatment. Finally, Lopez et al. (2008) in a multicenter, 12-week, open-label study, assessed the efficacy of donepezil in 106 Hispanic patients. The NPI subdomain “apathy/indifference” showed statistically significant improvement. In a retrospective clinical cohort study (Okayama Memantine Study) by Matsuzono et al. (2015a), the clinical effects of combination therapy of donepezil plus memantine (n - 61) or galantamine plus memantine (n - 53) in patients with AD were investigated. The authors concluded that the combination therapy of galantamine plus memantine may be better for cognitive aspects of the older patients with AD, and donepezil plus memantine may be better for apathy in the older patients with AD. In a following retrospective clinical cohort study, Okayama Late Dementia Study, Matsuzono et al. (2015b) examined the effects of monotherapy donepezil (n - 55), galantamine (n - 222), rivastigmine (n - 63), or memantine (n - 33) in older patients with AD. Apathy scale scores were well preserved until 12 months for all four drugs.

### Galantamine

Six studies (four RCTs and two open-label studies) were found to assess the efficacy of galantamine, an AChEI and nicotine modulator, in the treatment of apathy in AD. Apathy was assessed in five studies with NPI and in one with an 11-item behavior assessment scale. All studies except one demonstrated at least some clinical benefit in apathy scores after treatment.

*Randomized controlled trials*: Four RCTs were found to assess the efficacy of galantamine in the treatment of apathy in AD. Tariot et al. (2000) conducted a 5-month RCT to assess the efficacy of galantamine in 978 patients. Unlike the placebo group, the galantamine-treated groups did not show deterioration in behavioral symptoms, as indicated by NPI scores. Rockwood et al. (2001) conducted a 3-month, multicenter RCT in 386 patients. Behavioral symptoms as assessed by NPI did not change significantly neither in placebo nor in the galantamine-treated group. Moreover, significantly higher adverse events, such as nausea 13%, vomiting 6%, dizziness 5%, and anorexia 4%, occurred in the treatment group. In a multicenter 6-month RCT conducted by Erkinjuntti et al. (2002), the efficacy of galantamine was assessed in 457 patients. NPI apathy scores improved significantly from baseline in the galantamine-treated group versus placebo. Cummings et al. (2004) investigated the efficacy of galantamine with a 21-week, multicenter RCT in 978 patients. An observed case analysis of patients without specific behavioral symptoms at baseline revealed significantly less emergence of apathy in galantamine treated patients.

*Open-label studies*: Monsch et al. (2004) conducted a 3-month, open-label, multicenter study in Switzerland in which the effect of galantamine was assessed in 124 patients. A 27% reduction in NPI apathy score was observed after treatment. Brodaty et al. (2006) found that 6 months of galantamine treatment stabilized or improved apathy scores in many of the 345 participants.

### Memantine

Six studies (four RCTs, one open-label study, and one post marketing surveillance study) were found to assess the efficacy of memantine, an N-methyl-D-aspartate (NMDA) receptor antagonist, in the treatment of apathy in AD. Apathy was assessed in three studies with NPI, one with Sandoz Clinical Assessment–Geriatric (SCAG) scale, one with the Nurses’ Observation Scale for Inpatient Evaluation (NOSIE), and the last with Behavioral Rating Scale for geriatric patients (BGP) scale. One study had negative results, while the others demonstrated at least some benefit in apathy scores.

*Randomized controlled trials*: Pantev et al. (1993) conducted a 4-week RCT to assess the efficacy of memantine in 60 patients. After treatment, improvement in apathy scores (as evaluated by SCAG and NOSIE scales) was observed. Winblad and Poritis (1999) investigated the efficacy of memantine in 151 patients. Memantine-treated patients showed significant improvement in the hobbies/interest BGP subscale. Cummings et al. (2006b), in an exploratory analysis of a 24-week RCT, compared memantine treatment with placebo in 404 patients on stable donepezil treatment. Analyses of the 12 NPI domains revealed significant effects in favor of memantine on agitation/aggression, eating/appetite, and irritability but not on apathy. In a recent RCT by Zhou et al. (2019), 80 patients with moderate AD randomly received memantine plus either citalopram (n = 40, study group) or placebo (n = 40, control group) in a 12week period. Apathy received NPI lower scores in participants who received memantine combined with citalopram vs. those before treatment.

*Open-label studies*: Schmidt et al. (2010) conducted a 16-week open label study to assess the efficacy of memantine in 53 patients. Improvement in the NPI apathy score (-11.3%) was observed after treatment. Postmarketing surveillance study: Clerici et al. (2012) in a postmarketing surveillance study of 399 memantine-treated patients, free of cholinergic medications, found that 49 patients showed improvement in the apathy scores.

### Tacrine

Three studies (one RCT and two open-label studies) were identified that assess the efficacy of tacrine in reducing apathy in patients with AD. Apathy was assessed in two studies with NPI and in one with the NOSIE and the BGP scale. One study had negative results, while the others demonstrated at least some benefit in the apathy scores. Tacrine is not in use due to its serious adverse effect profile (hepatotoxicity).

*Randomized controlled trials*: In an RCT conducted by Ahlin et al. (1991), the efficacy of tacrine (75–150 mg/day) versus placebo was investigated in 15 patients over 9 weeks. No difference in “interest” on NOSIE scale was observed after treatment with tacrine.

*Open-label studies*: In two open-label trials (Kaufer et al., 1996; Kaufer et al., 1998), the efficacy of tacrine was assessed in 28 and 40 patients, respectively. In the first one (Kaufer et al., 1996), the improvement in NPI apathy score did not reach significance, while in the second one, apathy was significantly reduced (Kaufer et al., 1998).

### Metrifonate

*Randomized controlled trials*: Two RCTs were found to assess the efficacy of metrifonate in the treatment of apathy in AD. Apathy was assessed in both studies with NPI. Both studies demonstrated benefit in apathy scores after metrifonate use. Metrifonate is not in use due to its serious adverse effect profile (respiratory paralysis and problems with neuromuscular transmission). In the 26-week RCT by Kaufer (1998), 393 patients participated. Statistically significant mean change difference was found in the NPI apathy score after treatment with metrifonate. In an RCT conducted by Dubois et al. (1999), 605 patients participated. Significant improvement in apathy was observed after treatment with metrifonate.

### Rivastigmine

*Open-label studies*: Eight open-label studies were identified to assess the efficacy of rivastigmine in the treatment of apathy in AD. Apathy was assessed in five studies with NPI and in three studies with the abbreviated Clinician’s Global Impression of Change (CGIc). All the studies demonstrated at least some benefit in apathy scores after treatment with rivastigmine. In a 6-month open-label study by Dartigues et al. (2002), 696 patients participated. Significant improvement was observed in the NPI apathy score after 12 weeks of treatment with rivastigmine but not after 6 months. Hatoum et al. (2005) conducted a prospective, multicenter, 52-week open-label study in 173 patients. The higher the daily dose of rivastigmine, the less likely were the patients to experience apathy. In a 12-week open-label study by Bullock et al. (2001) [first part of Cummings et al. (2005)], 173 patients participated. Improvements were noted in the mean NPI apathy score after treatment with rivastigmine. Cummings et al. (2005) conducted a prospective, 26-week, open-label study assessing the effects of rivastigmine (3–12 mg/day) in 173 nursing home residents. Significant improvement in NPI apathy scores was observed. In a prospective, multicenter 26-week open-label study (Aupperle et al., 2004; Edwards et al., 2005), extension to the 26-week study by Cummings et al. (2005), 72 patients participated. A 39.3% reduction in apathy symptoms from baseline was found (P = 0.008). Gauthier et al. (2006) conducted an observational open-label study in 2,633 patients. The effect of rivastigmine in apathy (assessed with CGIc) was reported to be clinically significant. In a study, Gauthier et al. (2007), in 2,119 patients, found that 62.6% of rivastigmine-treated patients experienced improvements in apathy. Finally, Gauthier et al. (2010) in an open-label, multicenter study assessed the efficacy of rivastigmine in 3,800 patients. The proportions of patients improving versus deteriorating at month 6 were 42.8% versus 7.2% for apathy.

### Ginkgo Biloba

*Randomized controlled trials*: Three studies (three RCTs) were found to assess the efficacy of ginkgo biloba in the treatment of apathy in AD. Scripnikov et al. (2007), Bachinskaya et al. (2011), and Ihl et al. (2011) found significant improvement in NPI apathy scores after treatment with ginkgo biloba in 400, 404, and 410 patients, respectively.

### Methylphenidate

Apathy seems to be related to some degree of dopaminergic neuronal loss; enhancing of dopaminergic transmission with methylphenidate may partially reverse the effects of apathy (Galynker et al., 1997; Hermann et al., 2008; Padala et al., 2010; Rosenberg et al., 2013; Lanctôt et al., 2014; Padala et al., 2017).

Five studies (three RCTs and two open-label studies) were found assessing the efficacy of methylphenidate in the treatment of apathy in AD. Apathy was measured in two studies with Apathy Evaluation Scale (AES), in one study with AES and NPI, in one study with the NOSIE, and in one study with the Scale for the Assessment of Negative Symptoms (SANS). All the studies demonstrated at least some benefit in apathy scores after treatment with methylphenidate; adverse events (delusions, irritability, etc.) were reported in some patients.

*Randomized controlled trials*: Hermann et al. (2008) in a 5-week crossover RCT, found that 13 apathetic patients demonstrated greater improvement after treatment with methylphenidate (P = 0.047). Two patients experienced serious adverse events (delusions, irritability, etc.), which resolved upon drug discontinuation. Rosenberg et al. (2013) in a 6-week RCT, assessed the efficacy of methylphenidate 20 mg/day in 60 apathetic patients and found that NPI apathy score improvement was significantly greater on methylphenidate versus placebo (P = 0.02); only 17 of completers had improved apathy scores (Lanctôt et al., 2014). Padala et al. (2017) in a12-week, RCT assessed the efficacy of methylphenidate in 60 community-dwelling veterans with mild AD; they found that AES-C apathy score improvement was significantly greater on methylphenidate versus placebo (P < 0.001).

*Open-label studies*: Galynker et al. (1997) conducted a pilot study in order to assess the efficacy of methylphenidate (5–20 mg/day) on negative symptoms in 27 patients. Significant improvement was observed in SANS from baseline. Two patients dropped out due to emergence of agitation and psychosis. Padala et al. (2010) in a 12-week study with 23 patients, observed significant improvement in AES apathy scores after treatment with methylphenidate.

### Modafinil

Frakey et al. (2012) conducted an 8-week RCT in 23 patients being on a stable dose of an AChEI to estimate the efficacy of modafinil. Modafinil treatment did not result in significant additional reductions in the Frontal Systems Behavior Scale apathy scores. However, reductions in perceived apathetic symptomatology were correlated with reductions in reported caregiver burden.

### Deanol

One open-label study assessed the effect of deanol (Ferris et al., 1977), a choline precursor, to treat apathy in 14 patients. The total score on the SCAG scale was lowered by the third week (P < 0.01), as a result of reduced depression, irritability, anxiety, and increased motivation initiative.

### Antidepressants

Although often prescribed (Benoit et al., 2008), antidepressants do not significantly improve apathy in people with AD (Tariot et al., 1987; Nyth et al., 1992; Freedman et al., 1998; Pollock et al., 2002; Lyketsos et al., 2003; Siddique et al., 2009; Porsteinsson et al., 2014) with the exception of a recent RCT in which memantine and citalopram were combined (Zhou et al., 2019). In a RCT by Siddique et al. (2009) in which citalopram was prescribed for irritability, a large decrease in irritability of non-depressed patients was followed with a non-significant decrease in apathy. It is of interest that an increase in apathy was reported with the use of selective serotonin reuptake inhibitors (SSRIs) in depressed elderly patients (Wongpakaran et al., 2007). However, findings were not specific for patients with AD.

### Atypical Antipsychotics

Atypical antipsychotics have shown favorable therapeutic response in apathetic patients with AD (Oberholzer et al., 1992; Negron and Reichman, 2000; De Deyn et al., 2004; Onor et al., 2007). However, extensive use of antipsychotics in dementia is not recommended since it is associated with serious adverse effects (Food and Drug Administration, 2005).

### Conventional Antipsychotics

There are no studies using conventional antipsychotics to treat apathy specifically for patients with AD; these are mainly older studies in patients with various forms of dementia (Barton and Hurst, 1966; Birkett et al., 1972; Kirven and Montero, 1973; Cahn and Diesfeldt, 1973; Gotestam et al., 1981; Barnes et al., 1982; Petrie et al., 1982; Lovett et al., 1987; Pollock et al., 2002). Furthermore, conventional antipsychotics seem to carry greater risks for patients with AD in terms of extrapyramidal symptoms (EPS), cardiac arrhythmias, and overall mortality (Trifiro et al., 2009; Trifiro et al., 2014).

### Other Psychotropic Compounds

In two studies, a calcium antagonist nimodepine (Ban et al., 1990; Pantoni et al., 1996) was used to treat apathy; these studies included patients with cognitive impairment and various forms of dementia. In one study, pentofylline (Bayer et al., 1996) was used to treat apathy in patients with multi-infarct dementia. For what concerns the use of anticonvulsants, two small studies, one RCT involving valproate in patients with senile dementia (Sival et al., 2002) and one open study involving gabapentin in patients with probable AD (Moretti et al., 2003), yielded negative results.

### Evidence From *Post Hoc*, and Pooled Data Analyses

#### Metrifonate

Cummings et al. (2001) did a retrospective analysis on 672 patients, in which NPI data from two 26-week lasting RCTs (Morris et al., 1998; Raskind et al., 1999) were pooled together (**Table 4**). At 26 weeks, metrifonate-treated patients had significantly reduced apathy score versus placebo.

**Table 4:** Meta-analyses, *post hoc* and pooled data analyses of agents reporting on apathy outcomes.

Study	N	Treatment	Duration considered (weeks)	Scale	Variable	Results/Effect size	Outcome	Comments
Waldemar et al. (2011)	490 (2 RCTs)	***Donepezil***						
		Feldman et al. (2001)	24	12-item NPI	First emergence of an apathy composite score (frequency x severity) ≥ 3.	Significant difference favoring donepezil (*p* = 0.01. Odds Ratio: 1.7)	Positive	- Female were significantly more in the donepezil group (*p *= 0.01) - Mild-to-moderate disease stage
		Winblad et al. (2001)	24	10-item NPI				
Herrmann et al. (2005)	2.033 (3 RCTs)	***Galantamine***						
		Rockwood et al. (2001)	12	NPI	A ≥30% change in apathy individual score considered as clinically relevant	-0.03^a^	Negative	p = 0.28
		Tariot et al. (2000)	20					
		Data on file (Janssen-Ortho Inc.)	24					
Gauthier et al. (2008)	2.311 (6 RCTs)	***Memantine***						
		Peskind et al. (2006)	24	12-item NPI	- Symptom improvement: lower apathy score than in baseline. - Symptom emergence: appearance of new apathy symptom while absent at baseline.		Negative	Mild to severe disease stages
		Porsteinsson et al. (2008)	24. Patients were on stable dose of donepezil, rivastigmine, or galantamine					
		Bakchine and Loft (2008)	24					
		van Dyck et al. (2007)	24					
		Tariot et al. (2004)	24. Patients were on stable dose of donepezil					
		Reisberg et al. (2003)	28					
Cummings et al. (2001)	672 (2 RCTs)	***Metrifonate***						
		Morris et al. (1998)	26	NPI	>30% reduction in the total NPI score	15% treatment benefit (*P* = 0.02)	Positive	- MMSE: 10-26
		Raskind et al. (1999)	26					- NPI was secondary outcome measure in both studies
Ruthirakuhan et al. (2018)	6384 (21 RCTs – four included for meta-analysis)	***Methylphenidate***						
		Hermann et al. (2008)	5	AES	Difference in the change in AES means (SD) scores from baseline to week 5	-2.31 (5.11), Wilcoxon Z = -2.00, *P* = 0.045	Positive	- Significant difference revealed for AES apathy but not for NPI-apathy - Apathy improvement with methylphenidate can be predicted on the basis of attentional response (i.e., increased inattention) to D-amph challenge - Tolerability concerns
		Rosenberg et al. (2013)	6	AES	Difference in the change in AES scores from baseline to week 6	−2.5 95%CI(−6.5, 1.6) *P* = 0.23	Negative	- Adequately powered study
		Padala et al. (2017)	12	AES-C	Score on the AES-C	-9.9, 95%CI(-13.6, -6.2) p < 0.001	Positive	- Higher apathy scores in the methylphenidate group at baseline
		***Modafinil***						
		Frakey et al. (2012)	8	FrSBe	Post-treatment score on the FrSBe	*F*_1,20_ = 2.160, *P* = 0.157, η^2^ = 0.097	Negative	A placebo effect is hypothesized for significant decreases observed in both groups
Sepehry et al. (2017)	4864 (15 RCTs - eleven included for meta-analysis)	***Donepezil***						
		Gauthier et al. (2002)	24	NPI-apathy	Least squares means change from baseline score (±SE)	-2.58±0.69 (p < 0.0001 vs. placebo)	Positive	No significant differences in symptom emergence between groups
		Tariot et al. (2001)	24	NPI-NH	Drug-placebo differences in least squares means change from baseline scores		Negative	Primary outcome was the NPI total score
		Seltzer et al. (2004)	24	Shortened version of AES	Least squares means change from baseline scores		Negative	Apathy was secondary outcome
		***Galantamine***						
		Herrmann et al. (2005)	12, 20, 24	NPI	Mean changes in scores from baseline	-0.03^a^	Negative	- Post hoc analysis on pooled data from 3 multicenter, double blind RCTs (N = 2.033) differing in durations, doses, formulations of galantamine, and the dosing/titration schedules used. - Apathy was secondary outcome
		***Memantine***						
		Cummings et al. (2006b)	24	NPI	Least-squares means change from baseline on total NPI		Negative	- Apathy was secondary outcome
		Gauthier et al. (2008)	See above					
		Herrmann et al. (2013)	24	NPI	Change from baseline in NPI total scores	Baseline-to-week-24 difference in means 1.23, 95%CI(−1.75, 4.21) P = 0.42	Negative	- Efficacy based on observed cases analysis - NPI total score was primary outcome and Apathy item score secondary
		Araki et al. (2014)	24	NPI	Least squares means change from baseline score (±SE)	Significant difference observed between the groups (*p* < 0.01)	Positive	- Small Sample - Multiple testing corrected results - A relationship between NIRS measured hemodynamic group changes and apathy was not reported
		***Modafinil***						
		Frakey et al. (2012)	See above					
		***Methylphenidate***						
		Hermann et al. (2008)	See above					
		Rosenberg et al. (2013)						

a Expressed as the Cohen’s Δ (mean change on apathy score divided by the pooled within-group standard deviation).

NPI, neuropsychiatric inventory; AES-C, Apathy Evaluation Scale – Clinician input; FrSBe, Frontal Systems Behavior Scale, AES, Apathy Evaluation scale; IFG, Inferior Frontal Gyrus; NIRS, Near Infrared Spectroscopy.

#### Donepezil

In a study by Waldemar et al. (2011)(**Table 4**), two RCTs (N = 490) were included (Feldman et al., 2001; Winblad et al., 2001). The authors proposed that donepezil may delay the onset of apathy in mild to moderate AD. However, both studies, although sharing design similarities, have used different effectiveness tools that do not specifically assess the clinical syndrome of apathy.

#### Galantamine

Herrmann et al. (2005) (**Table 4**) conducted a meta-analysis of three RCTs [Tariot et al., 2000; Rockwood et al., 2001; Data on file (Janssen-Ortho Inc)] in which the efficacy of galantamine was assessed in 2,033 patients. Patients treated with galantamine experienced a reduction in mean NPI apathy score from baseline; however, there was no significant difference between galantamine treated and placebo groups.

#### Memantine

Gauthier et al. (2005) analyzed the data of two RCTs (Reisberg et al., 2003; Cummings et al., 2006b) examining the efficacy of memantine in 656 patients. There was a trend for improvement in NPI apathy scores after treatment. Gauthier et al. (2008) (**Table 4**) conducted a pooled data analysis of six (24/28-week) RCTs including 2,311 patients (Reisberg et al., 2003; Tariot et al., 2004; Peskind et al., 2006; van Dyck et al., 2007; Bakchine and Loft, 2008; Porsteinsson et al., 2008). Apathy score did not differ significantly between memantine-treated and placebo groups.

## Discussion

### Principal Findings

We attempted to systematically review apathy treatments in AD across pharmacological treatment modalities including combined treatments. In many studies, apathy was not considered as the primary outcome measure (**Table 2**). Considering the amount of evidence, AChEIs may be the best available treatment choice for the pharmacological treatment of apathy. Reduced cholinergic tone has been argued for the presentation of apathy (Mega et al., 1997), and thus, AChEIs might prove helpful in this domain (Trinh et al., 2003; Cummings et al., 2008). Gingko biloba was also found to be effective (Scripnikov et al., 2007; Bachinskaya et al., 2011; Ihl et al., 2011). There was weaker evidence of efficacy for memantine; however, there are reports that memantine might be more effective in agitation and irritability (Cummings et al., 2008). Stimulants like methylphenidate alone or in combination with AChEIs have also been proven beneficial, and there is room for improvement in this category. Research groups might test hypotheses based on results from existing high-quality trials (Hermann et al., 2008; Rosenberg et al., 2013; Lanctôt et al., 2014). Atypical antipsychotics, on the other hand, cannot be used for long periods of time (Food and Drug Administration, 2005), while antidepressants (e.g., SSRIs, chlorophenylpiperazine, L-deprenyl) (Tariot et al., 1987; Nyth et al., 1992; Freedman et al., 1998; Pollock et al., 2002; Lyketsos et al., 2003; Siddique et al., 2009; Porsteinsson et al., 2014), with the exception of one study (Zhou et al., 2019), failed to improve patients’ apathy significantly.

### Strengths and Weaknesses

#### Appraisal of Methodological Quality of the Review

We reviewed a large body of evidence using flexible criteria to capture a more pragmatic picture on pharmacological treatment of apathy in AD. Although a certain amount of studies showed at least some benefit of various pharmacological interventions for apathetic symptoms, most of them reviewed were not designed to target apathy. In fact, the consistency of the widely used NPI-apathy item with more specific apathy scales, like AES, was reported to be problematic (Rosenberg et al., 2013). This, along with inconsistently reporting effect sizes and their parameters and the variety of psychometric tools used to assess apathy (sometimes involving scales not validated), would make a meta-analysis incomplete and/or misleading. Therefore, we evaluated the quality of the reported evidence using two published semiquantitative methods.

#### Several Limitations Apply to This Review

Any conclusions drawn rely on the quality of the included studies, while an unknown number of studies that involve combined treatments might have been excluded by the review design of older reviews. The studies included in this review were methodologically heterogeneous and conducted mainly with AChEIs, while those conducted with cognitive enhancers incorporated smaller sample sizes. Furthermore, age and gender may potentially have influenced the pharmacological effects (Matsuzono et al., 2015b). While not so prone to publication bias, as apathy was the secondary outcome in more cases, selective reporting cannot be excluded.

### Relation to Other Reviews

There are only few reviews that specifically have focused on apathy outcomes in dementia and AD following the use of pharmacological interventions (Cummings et al., 2008; Drijgers et al., 2009; Berman et al., 2012; Theleritis et al., 2017). In their review, Drijgers et al. (2009) proposed that cholinesterase inhibitors and methylphenidate may be the best candidates for further study in the treatment of apathy in dementia. It is of interest that in a recent review by Sherman et al. (2018), the use of galantamine and risperidone was found to produce mild reductions of apathetic behaviors in patients with prodromal dementia.

The fact that in few studies apathy was the primary outcome measure led Ruthirakuhan et al. (2018) to define two objectives in their recent Cochrane systematic review on pharmacological interventions for apathy in AD, or mixed AD populations (**Table 2**). Objective 1 was the efficacy and safety, based on studies where clinically significant apathy (mean baseline NPI-apathy sub-score > 3) was the primary outcome, and Objective 2 was the effect of pharmacotherapies for other primary outcomes and apathy was a secondary outcome. Four studies in mild-to-moderate AD, three on 20 mg methylphenidate (Hermann et al., 2008; Rosenberg et al., 2013; Padala et al., 2017) and one on modafinil (Frakey et al., 2012), were employed for Objective 1. Based on AES score in three studies, methylphenidate was found to improve apathy compared to placebo [mean difference (MD) = -4.99, 95%CI (-9.55, -0.43), P = 0.03, n = 145, 3 studies; heterogeneity: *I^2^* = 83%)]. A subgroup analysis by treatment duration (cutoff, 12 weeks) revealed significant differences between subgroups (greater apathy changes in administration longer than 12 weeks). However, the level of evidence in studies that have used the AES was rated as low (i.e., there is limited confidence for the closeness of the estimated effect to the true, and thus the true effect may be substantially different from the estimated effect). Contributing factors, as suggested by the authors, were imprecision due to a wide 95% CI and the inconsistency, due to heterogeneity, in the effects on apathy in the three methylphenidate studies. Based on NPI-apathy score in two studies, methylphenidate appeared not to have a significant effect on apathy [MD = -0.08, 95%CI (-3.85, 3.69), P = 0.97, n = 85, 2 studies; heterogeneity: *I^2^* = 84%]. This inconsistency was attributed by the authors to the type of scales and the smaller total number of participants included in the analysis with NPI. There were no significant differences in adverse events between subgroups [Chi^2^(1) = 0.03, P = 0.85; heterogeneity: *I^2^* = 0%]. Again, the low quality rated evidence substantially affects the certainty in these results. For Objective 2, six studies with AChEIs in AD patients and clinically nonsignificant apathy were included. AChEIs may slightly improve apathy compared to placebo, and this effect is not influenced by disease severity. On the other hand, discontinuation of AChEIs (1 study) revealed no significant improvement, antipsychotics (two studies) worsened apathy, discontinuation of antipsychotics (one study) revealed a slightly significant improvement, while there was uncertainty for what concerns antidepressants (two studies) due to very low evidence quality.

In a previous systematic review followed by meta-analysis (Sepehry et al., 2017) (**Table 2**), 15 RCTs were included of which 11 entered the meta-analysis. Studies on three drug classes were analyzed as passed the three-study-inclusion cutoff, namely, four on AChEIs (three on donepezil and one on galantamine), four on memantine, and three on stimulants (two on methylphenidate and one on modafinil). In 9 of 11 studies, the NPI-apathy item was used to measure apathy, while AES (in a donepezil study) and Frontal Systems Behavior Scale (FrSBe) (in the modafinil study) were also used. No significant treatment effect was estimated for the cognitive enhancers [Hedges’g = -0.055, 95%CI (-0.322, 0.213), P = 0.687; heterogeneity: Q-value = 17.378, P = 0.001, I2 = 82.737], and small and nonsignificant effects were estimated for memantine [Hedges’ g = 0.092, 95%CI (-0.134, 0.318), P = 0.423; heterogeneity: Q-value = 11.425, P = 0.010, *I^2^* = 73.742] and the stimulants [Hedges’ g = -0.063, 95%CI (-1.067, 0.941), P = 0.903; heterogeneity: Q-value = 12.486; P = 0.002; *I^2^* = 83.982]. Besides the drugs’ ineffectiveness, the lack of a significant drug effect was attributed, by the authors, to the variable disease state in the studies, the small sample size in the psychostimulant studies, the heterogeneity in the study design, as well as the review methodology (means aggregate was calculated from a small number of studies in each drug class, which does not allow to control for the above factors, while various studies had been excluded by the review design).

In the review by Harrison et al. (2016) on pharmacological trials targeting apathy in various dementia types, 24 studies (10 in AD, 6 in FTLD, 4 in dementia or probable dementia and MCI, 1 in PDD, 1 in LBD, and 2 in unspecified dementia) were included. Studies from the last 3 years were included for review, and the authors conclude that the evidence is not convincing. However, benefits from AChEIs and memantine seen in earlier studies did not appear in more recent studies. A model of therapies is proposed that act in a synergistic manner, as well as stepped approaches starting from psychosocial interventions. In the review by Ruthirakuhan et al. (2018), methylphenidate was found to demonstrate a benefit for apathy and slightly improve cognition and functional performance in people with AD.

### Implications

#### Strengths and Weaknesses of the Evidence Included in the Review

A wide range of agents are included in this review. It appears that combined treatments (Chapman et al., 2004; Niu et al., 2010; Zhou et al., 2019) might be of greater benefit compared with monotherapies, and future studies should address this research topic more rigorously. Variable sensitivity of apathy scales may confound results and their interpretation.

#### Direction and Magnitude of Effects Observed in the Included Studies

Although a number of compounds proved beneficial, effect sizes were small. When symptoms resist and interfere with significant life domains, AChEIs and methylphenidate seem to be the first pharmacological choice, followed by gingko biloba. Only effect sizes provided from previous meta-analyses are reported.

### Practical Implications for Clinicians and Policy Makers.

#### Implications for Practice

Poorer results in treating apathy in AD, from the concomitant use of psychoactive medication, may be indicative of greater disease severity. Furthermore, ethical constraints apply when testing drugs for apathy in AD (e.g., the concomitant use of AChEIs). Last but not least, drugs may be acting differently depending on doses, age, type, and stage of neurodegeneration and probably the different apathy syndromes.

#### Implications for Research

Apathy is understudied and a work in progress as social interaction was incorporated in the recently updated diagnostic criteria for apathy (Robert et al., 2018). Indeed, psycho-social factors are well recognized and described in motivation research and goal-directed behavior (e.g., Deci, 1971). Studies should set up their design so that they facilitate symptom expression and engagement (individualized treatments, environmental and cultural considerations, preferences, psycho-education, etc.). Currently, the lack of standard and widely accepted tools concerning apathy differentially adds to studies’ heterogeneity and influences outcomes and their appraisal. It is worth mentioning that in a systematic review by Radakovic et al. (2015), among the highest quality apathy scales in AD were the DAIR and the AES–Clinical version, while in a review by Clarke et al. (2011), the most psychometrically robust measures for assessing apathy across any disease population appeared to be the AES and the apathy subscale of the NPI; while for patients with AD, the DAIR was found to be the most reliable and valid apathy measure. Moreover, the Lille Apathy Rating Scale (LARS, Sockeel et al., 2006) has been shown to assess emotional, cognitive, and behavioral aspects of apathy independently of depression, with high concurrent validity in relation to AES global scores and with reference to expert clinician categorization of syndrome severity.

Due to heterogeneity of the sample populations and the syndromic nature of apathy, its symptoms rather form clusters within different NPI symptoms, which are consistent across studies defining potential subsyndromes (Canevelli et al., 2013). In this context, recommendations on the design of clinical trials on apathy have recently been published (Cummings et al., 2015). Problematic allocation concealment and non-double-blind design may positively bias an effect by 41% and 17%, respectively (Schulz et al., 1995). Endorsement of CONsolidated Standards of Reporting Trials statement could substantially confer to completeness of the trials while quantification strategies in general are of great need. Clinical trials implementing combination strategies are of particular interest. Furthermore, different incident neuropsychiatric symptom profiles in relation to different vascular pathology (small vs. large vessel cerebrovascular disease) can inform and guide relevant studies (Staekenborg et al., 2010). It would also be interesting to investigate health outcomes and further individual implications by treatments administered for longer periods against the cost.

## Conclusion

We argue for informed treatments that are frequently combinations of therapeutic interventions administered in well-characterized patient groups based on rigorously designed RCTs with intervention outcomes assessed in long terms. It is, thus, fundamental to determine quantified markers and models of apathy (behavioral, social, imaging, computational, etc.) (Theleritis et al., 2014) after developing, validating, and standardizing diagnostics, interventions, and measures to effectively target apathy, while reconciling existing paradigms of specific apathy models and clinical research (Chong et al., 2016; Chong and Husain, 2016). Kales et al. (2014, 2015), for example, proposed that the “Describe, Investigate, Create, Evaluate’’ approach may enable clinicians to choose optimal treatment plans for the management of neuropsychiatric symptoms by considering conjointly the role of specific nonpharmacological, medical, and pharmacological treatment (Lanctôt et al., 2017; Theleritis et al., 2017; Theleritis et al., 2018). Concerning the measures for apathy, validation of the tools by the different apathy components may be of particular interest (Nobis and Husain, 2018). Incorporation of the “social interaction” term in the recent revision of diagnostic criteria for apathy (Robert et al., 2018) may aid developing more complete models and more effective treatments for apathy. Moreover, lifestyle modifications and context interventions, including exercise, leisure activities, cognitive stimulation, and social activities, might be effective for the prevention of apathy and MCI progression (Rosenberg and Lyketsos, 2008). All the above along with the accumulated neurobiological evidence (e.g., neuroimaging) and the emerging models (e.g., behavioral, computational) for apathy will help to identify target populations most likely responding to specific treatments.

## Author Contributions

All three authors have gone through all the abstracts; when there was disagreement between the 3 authors, the issue was resolved by a consensus meeting with the last author. The first two authors have written the review under the supervision of the last author.

## Conflict of Interest

The authors declare that the research was conducted in the absence of any commercial or financial relationships that could be construed as a potential conflict of interest.

## References

[B1] Ahlin A. Nyback H. Junthe T. Ohman G. Nordgren I. (1991). Tetrahydroaminoacridine in Alzheimer’s dementia: clinical and biochemical results of a double-blind crossover trial. Hum. Psychopharmacol. 6 (2), 109–118. 10.1002/hup.470060205

[B2] Araki T. Wake R. Miyaoka T. Kawakami K. Nagahama M. Furuya M. (2014). The effects of combine treatment of memantine and donepezil on Alzheimer’s disease patients and its relationship with cerebral blood flow in the prefrontal area. Int J. Geriatr. Psychiatry 29, 881–889. 10.1002/gps.4074 24436135

[B3] Aupperle P. M. Koumaras B. Chen M. Rabinowicz A. Mirski D. (2004). Long-term effects of rivastigmine treatment on neuropsychiatric and behavioral disturbances in nursing home residents with moderate to severe Alzheimer’s disease: results of a 52-week open label study. Curr. Med. Res. Opin. 20 (10), 1605–1612. 10.1185/030079904125004204 15462693

[B4] Bachinskaya N. Hoerr R. Ihl R. (2011). Alleviating neuropsychiatric symptoms in dementia: the effects of ginkgo biloba extract EGb 761. Findings from a randomized controlled trial. Neuropsychiatry Dis. Treat. 7, 209–215. 10.2147/NDT.S18741 PMC3090284 21573082

[B5] Bakchine S. Loft H. (2008). Memantine treatment in patients with mild to moderate Alzheimer’s disease: results of a randomised, doubleblind, placebo-controlled 6-month study. J. Alzheimers Dis. 13 (1), 97–107. 10.3233/JAD-2008-13110 18334761

[B6] Ban T. A. Morey L. Aguglia E. Azzarelli O. Balsano F. Marigliano V. (1990). Nimodipine in the treatment of old age dementias. Prog. Neuropsychopharmacol. Biol. Psychiatry 14, 525–551. 10.1016/0278-5846(90)90005-2 2236581

[B7] Barak Y. Bodner E. Zemishlani H. Mirecki I. Aizenberg D. (2001). Donepezil for the treatment of behavioral disturbances in Alzheimer’s disease: a 6-month open trial. Arch. Gerontol. Geriatr. 33 (3), 237–241. 10.1016/S0167-4943(01)00187-X 15374020

[B8] Barnes R. Veith R. Okimoto J. Raskind M. Gumbrecht G. (1982). Efficacy of antipsychotic medications in behaviorally disturbed dementia patients. Am. J. Psychiatry 139, 1170–1174. 10.1176/ajp.139.9.1170 7114310

[B9] Barton R. Hurst L. (1966). Unnecessary use of tranquilizers in elderly patients. Br. J. Psychiatry 112, 989–990. 10.1192/bjp.112.491.989 5339369

[B10] Bayer A. J. Bokonjic R. Booya N. H. Demarin V. Ersmark B. Fairbairn A. F. (1996). European pentoxifylline multi-infarct dementia study. Eur. Neurol. 36, 315–321. 10.1159/000117279 8864715

[B11] Benoit M. Dygai I. Migneco O. Robert P. H. Bertogliati C. Darcourt J. (1999). Behavioral and psychological symptoms in Alzheimer’s disease. Relation between apathy and regional cerebral perfusion. Dement. Geriatr. Cogn. Disord. 10 (6), 511–517. 10.1159/000017198 10559568

[B12] Benoit M. Andrieu S. Lechowski L. Gillette-Guyonnet S. Robert P. H. Vellas B. (2008). Apathy and depression in Alzheimer’s disease are associated with functional deficit and psychotropic prescription. Int. J. Geriatr. Psychiatry 23 (4), 409–414. 10.1002/gps.1895 17918770

[B13] Berman K. Brodaty H. Withall A. Seeher K. (2012). Pharmacologic treatment of apathy in dementia. Am. J. Geriatr. Psychiatry 20 (2), 104–122. 10.1097/JGP.0b013e31822001a6 21841459

[B14] Birkett D. P. Hirschfield W. Simpson G. M. (1972). Thiothixene in the treatment of diseases of the senium. Curr. Ther. Res. Clin. Exp. 14, 775–779. 4629816

[B15] Brodaty H. Woodward M. Boundy K. Barnes N. Allen G. Investigators, NATURE (2006). A naturalistic study of galantamine for Alzheimer’s disease. CNS Drugs 20 (11), 935–943. 10.2165/00023210-200620110-00006 17044730

[B16] Bullock R. Moulias R. Steinwachs K. C. Cicin-Sain A. Spiegel R. (2001). Effects of rivastigmine on behavioural symptoms in nursing home patients with Alzheimer’s disease. Int. Psychogeriatry 13 (suppl 2), 242. 10.1017/S1041610202008207

[B17] Cahn L. A. Diesfeldt H. F. (1973). The use of neuroleptics in the treatment of dementia in old age. A critical analysis with reference to an experiment with a long-acting oral neuroleptic (penfluridol Janssen). Psychiatry Neurol. Neurochir. 76, 411–420. 4592700

[B18] Canevelli M. Adali N. Voisin T. Soto M. E. Bruno G. Cesari M. (2013). Behavioral and psychological subsyndromes in Alzheimer’s disease using the neuropsychiatric inventory. Int J. Geriatr. Psychiatry 28 (8), 795–803. 10.1002/gps.3904 23147419

[B19] Chapman S. B. Weiner M. F. Rackley A. Hynan L. S. Zientz J. (2004). Effects of cognitive-communication stimulation for Alzheimer’s disease patients treated with donepezil. J. Speech Lang. Hear. Res. 47 (5), 1149–1163. 10.1044/1092-4388(2004/085) 15603468

[B20] Chong T. T.-J. Bonnelle V. Husain M. (2016). “Chapter 4—Quantifying motivation with effort-based decision-making paradigms in health and disease,” in Progress in Brain Research, vol. 229 Eds. Studer B. Knecht S. (Amsterdam: Elsevier), 71–100. 10.1016/bs.pbr.2016.05.002 27926453

[B21] Chong T. T.-J. Husain M. (2016). “Chapter 17-The role of dopamine in the pathophysiology and treatment of apathy,” in Progress in Brain Research, vol. 229 Eds. Studer B. Knecht S. (Amsterdam: Elsevier), 389–426. 10.1016/bs.pbr.2016.05.007 27926449

[B22] Clarke D. E. Ko J. Y. Kuhl E. A. van Reekum R. Salvador R. Marin R. S. (2011). Are the available apathy measures reliable and valid? A review of the psychometric evidence. J. Psychosom. Res. 70 (1), 73–97. 10.1016/j.jpsychores.2010.01.012 21193104 PMC3902773

[B23] Clarke D. E. Ko J. Y. Lyketsos C. Rebok G. W. Eaton W. W. (2010). Apathy and cognitive and functional decline in community dwelling older adults: results from the Baltimore ECA longitudinal study. Int. Psychogeriatry 22 (5), 819–829. 10.1017/S1041610209991402 PMC2893259 20478091

[B24] Clerici F. Vanacore N. Elia A. Spila-Alegiani S. Pomati S. Da Cas R. (2012). Memantine effects on behaviour in moderately severe to severe Alzheimer’s disease: a post-marketing surveillance study. Neurol. Sci. 33 (1), 23–31. 10.1007/s10072-011-0618-0 21584738

[B25] Cummings J. L. (1997). The Neuropsychiatric Inventory: assessing psychopathology in dementia patients. Neurology 48 (suppl6), s10–s16. 10.1212/WNL.48.5_Suppl_6.10S 9153155

[B26] Cummings J. L. McRae T. Zhang R. Donepezil-Sertraline Study Group (2006a). Effects of donepezil on neuropsychiatric symptoms in patients with dementia and severe behavioral disorders. Am. J. Geriatr. Psychiatry 14 (7), 605–612. 10.1097/01.JGP.0000221293.91312.d3 16816014

[B27] Cummings J. L. Schneider L. Tariot P. N. Kershaw P. R. Yuan W. (2004). Reduction of behavioral disturbances and caregiver distress by galantamine in patients with Alzheimer’s disease. Am. J. Psychiatry 161 (3), 532–538. 10.1176/appi.ajp.161.3.532 14992980

[B28] Cummings J. L. Schneider E. Tariot P. N. Graham S. M. Memantine MEM-MD-02 Study Group (2006b). Behavioral effects of memantine in Alzheimer disease patients receiving donepezil treatment. Neurology 67 (1), 57–63. 10.1212/01.wnl.0000223333.42368.f1 16832078

[B29] Cummings J. L. Koumaras B. Chen M. Mirski D. Rivastigmine Nursing Home Study Team (2005). Rivastigmine Nursing Home Study Team. Effects of rivastigmine treatment on the neuropsychiatric and behavioral disturbances of nursing home residents with moderate to severe probable Alzheimer’s disease: a 26-week, multicenter, open-label study. Am. J. Geriatr. Pharmacother. 3 (3), 137–148. 10.1016/S1543-5946(05)80020-0 16257816

[B30] Cummings J. L. Nadel A. Masterman D. Cyrus P. A. (2001). Efficacy of metrifonate in improving the psychiatric and behavioral disturbances of patients with Alzheimer’s disease. J. Geriatr. Psychiatry Neurol. 14 (2), 101–108. 10.1177/089198870101400211 11419566

[B31] Cummings J. L. Mackell J. Kaufer D. (2008). Behavioral effects of current Alzheimer’s disease treatments: a descriptive review. Alzheimers Dement. 4 (1), 49–60. 10.1016/j.jalz.2007.10.011 18631950

[B32] Cummings J. Friedman J. H. Garibaldi G. Jones M. Macfadden W. Marsh L. (2015). Apathy in neurodegenerative diseases: recommendations on the design of clinical trials. J. Geriatr. Psychiatry Neurol. 28 (3), 159–173. 10.1177/0891988715573534 25809634

[B33] Dartigues J. Goulley F. Bourdeix I. Péré J. J. Barberger-Gateau P. (2002). Rivastigmine in current clinical practice in patients with mild to moderate Alzheimer’s disease [in French]. Rev. Neurol. 158 (8–9), 807–812. RN-09-2002-158-8-9-0035-3787 101019-ART4 12386525

[B34] Deci E. L. (1971). Effects of externally mediated rewards on intrinsic motivation. J. Pers. Soc. Psychol. 18, 105–115. 10.1037/h0030644

[B35] De Deyn P. P. Carrasco M. M. Deberdt W. Jeandel C. Hay D. P. Feldman P. D. (2004). Olanzapine versus placebo in the treatment of psychosis with or without associated behavioral disturbances in patients with Alzheimer’s disease. Int. J. Geriatr. Psychiatry 19 (2), 115–126. 10.1002/gps.1032 14758577

[B36] Drijgers R. L. Verhey F. R. Leentjens A. F. Ko¨hler S. Aalten P. (2011). Neuropsychological correlates of apathy in mild cognitive impairment and Alzheimer’s disease: the role of executive functioning. Int. Psychogeriatry 23 (8), 1327–1333. 10.1017/S1041610211001037 21729413

[B37] Drijgers R. L. Aalten P. Winogrodzka A. Verhey F. R. Leentjens A. F. (2009). Pharmacological treatment of apathy in neurodegenerative diseases: a systematic review. Dement. Geriatr. Cogn. Disord. 28 (1), 13–22. 10.1159/000228840 19609082

[B38] Dubois B. McKeith I. Orgogozo J. M. Collins O. Meulien D. (1999). A multicentre, randomized, double-blind, placebo-controlled study to evaluate the efficacy, tolerability and safety of two doses of metrifonate in patients with mild-to-moderate Alzheimer’s disease: the MALT study. Int. J. Geriatr. Psychiatry 14 (11), 973–982. 10.1002/(SICI)1099-1166(199911)14:11<936::AID-GPS39>3.0.CO;2-1 10556869

[B39] Edwards K. Koumaras B. Chen M. Gunay I. Mirski D. Rivastigmine Nursing Home Study Team (2005). Long-term effects of rivastigmine treatment on the need for psychotropic medications in nursing home patients with Alzheimer’s disease: results of a 52-week open-label study. Clin. Drug Invest. 25 (8), 507–515. 10.2165/00044011-200525080-00003 17532694

[B40] Erkinjuntti T. Kurz A. Gauthier S. Bullock R. Lilienfeld S. Damaraju C. V. (2002). Efficacy of galantamine in probable vascular dementia and Alzheimer’s disease combined with cerebrovascular disease: a randomised trial. Lancet 359 (9314), 1283–1290. 10.1016/S0140-6736(02)08267-3 11965273

[B41] Feldman H. Gauthier S. Hecker J. Vellas B. Subbiah P. Whalen E. (2001). Donepezil MSAD Study Investigators Group. A 24-week, randomized, double-blind study of donepezil in moderate to severe Alzheimer’s disease. Neurology 57 (4), 613–620. 10.1212/WNL.57.4.613 11524468

[B42] Feldman H. Gauthier S. Hecker J. Vellas B. Xu Y. Ieni J. R. (2005). Donepezil MSAD Study Investigators Group. Efficacy and safety of donepezil in patients with more severe Alzheimer’s disease: a subgroup analysis from a randomized placebo-controlled trial. Int. J. Geriatr. Psychiatry 20 (6), 559–569. 10.1002/gps.1325 15920715

[B43] Ferris S. H. Sathananthan G. Gershon S. Clark C. (1977). Senile dementia: treatment with deanol. J. Am. Geriatr. Soc. 25 (6), 241–244. 10.1111/j.1532-5415.1977.tb00407.x 864168

[B44] Food and Drug Administration (2005). Deaths with antipsychotics in elderly patients with behavioral disturbances. US Food and Drug Administration, FDA Public Health Advisory, Centre for Drug Evaluation and Research.

[B45] Frakey L. L. Salloway S. Buelow M. Malloy P. (2012). A randomized, double-blind, placebo-controlled trial of modafinil for the treatment of apathy in individuals with mild-to-moderate Alzheimer’s disease. J. Clin. Psychiatry 73 (6), 796–801. 10.4088/JCP.10m06708 22687392

[B46] Freedman M. Rewilak D. Xerri T. Cohen S. Gordon A. S. Shandling M. (1998). L-deprenyl in Alzheimer’s disease: cognitive and behavioral effects. Neurology 50 (3), 660–668. 10.1212/WNL.50.3.660 9521253

[B47] Galynker I. Ieronimo C. Miner C. Rosenblum J. Vilkas N. Rosenthal R. (1997). Methylphenidate treatment of negative symptoms in patients with dementia. J. Neuropsychiatry Clin. Neurosci. 9 (2), 231–239. 10.1176/jnp.9.2.231 9144102

[B48] Gauthier S. Feldman H. Hecker J. Vellas B. Ames D. Subbiah P. (2002). Donepezil MSAD Study Investigators Group. Efficacy of donepezil on behavioral symptoms in patients with moderate to severe Alzheimer’s disease. Int. Psychogeriatry 14 (4), 389–404. 10.1017/S104161020200858X 12670060

[B49] Gauthier S. Juby A. Morelli L. Rehel B. Schecter R. Investigators, EXTEND (2006). A large, naturalistic, community-based study of rivastigmine in mild-to-moderate AD: the EXTEND Study. Curr. Med. Res. Opin. 22 (11), 2251–2265. 10.1185/030079906X132749 17076986

[B50] Gauthier S. Juby A. Rehel B. Schecter R. EXTEND Investigators (2007). EXACT: rivastigmine improves high prevalence of attention deficits and mood and behavior symptoms in Alzheimer’s disease. Int. J. Clin. Pract. 61 (6), 886–895. 10.1111/j.1742-1241.2007.01387.x 17504350 PMC1974793

[B51] Gauthier S. Juby A. Dalziel W. Rehel B. Schecter R. Investigators, EXPLORE (2010). Effects of rivastigmine on common symptomatology of Alzheimer’s disease (EXPLORE). Curr. Med. Res. Opin. 26 (5), 1149–1160. 10.1185/03007991003688888 20230208

[B52] Gauthier S. Loft H. Cummings J. (2008). Improvement in behavioural symptoms in patients with moderate to severe Alzheimer’s disease by memantine: a pooled data analysis. Int. J. Geriatr. Psychiatry 23 (5), 537–545. 10.1002/gps.1949 18058838

[B53] Gauthier S. Wirth Y. Mobius H. J. (2005). Effects of memantine on behavioural symptoms in Alzheimer’s disease patients: an analysis of the Neuropsychiatric Inventory (NPI) data of two randomised, controlled studies. Int. J. Geriatr. Psychiatry 20 (5), 459–464. 10.1002/gps.1341 15852444

[B54] Gotestam K. G. Ljunghall S. Olsson B. (1981). A double-blind comparison of the effects of haloperidol and cis(Z)-clopenthixol in senile dementia. Acta Psychiatry Scand. 294 (Suppl), 46–53. 10.1111/j.1600-0447.1981.tb06213.x 7041519

[B55] Harrison F. Aerts L. Brodaty H (2016). Apathy in dementia: systematic review of recent evidence on pharmacological treatments. Curr. Psychiatry. Rep. 18 (11), 103. 10.1007/s11920-016-0737-7 27726067

[B56] Harvey L. Herbert R. Crosbie J. (2002). Does stretch induce lasting increases in joint ROM? A systematic review. Physiother. Res. Int. 7 (1), 1–13. 10.1002/pri.236 11992980

[B57] Hatoum H. T. Lin S. J. Arcona S. Thomas S. K. Koumaras B. Mirski D. (2005). The use of the occupational disruptiveness scale of the neuropsychiatric inventory-nursing home version to measure the impact of rivastigmine on the disruptive behavior of nursing home residents with Alzheimer’s disease. J. Am. Med. Dir. Assoc. 6 (4), 238–245. 10.1016/j.jamda.2005.04.003 16005409

[B58] Hermann N. Rothenburg L. S. Black S. Ryan M. Liu B. A. Busto U. E. (2008). Methylphenidate for the treatment of apathy in Alzheimer disease: prediction of response using dextroamphetamine. J. Clin. Psychopharmacol. 28 (3), 296–301. 10.1097/JCP.0b013e318172b479 18480686

[B59] Herrmann N. Rabheru K. Wang J. Binder C. (2005). Galantamine treatment of problematic behavior in Alzheimer’s disease: post-hoc analysis of pooled data from three large trials. Am J Geriatr Psychiatry 13 (6), 527–534. 10.1097/00019442-200506000-00012 15956273

[B60] Herrmann N. Gauthier S. Boneva N. Lemming O. M. 10158 Investigators (2013). A randomized, double-blind, placebo-controlled trial of memantine in a behaviorally enriched sample of patients with moderate-to-severe Alzheimer’s disease. Int. Psychogeriatry 25, 919–927. 10.1017/S1041610213000239 23472619

[B61] Holmes C. Wilkinson D. Dean C. Vethanayagam S. Olivieri S. Langley A. (2004). The efficacy of donepezilin the treatment of neuropsychiatric symptoms in Alzheimer disease. Neurology 63 (2), 214–219. 10.1212/01.WNL.0000129990.32253.7B 15277611

[B62] Ihl R. Bachinskaya N. Korczyn A. D. Vakhapova V. Tribanek M. Hoerr R. (2011). Efficacy and safety of a once-daily formulation of ginkgo biloba extract EGb 761 in dementia with neuropsychiatric features: a randomized controlled trial. Int. J. Geriatr. Psychiatry 26 (11), 1186–1194. 10.1002/gps.2662 21140383

[B63] Kales H. C. Gitlin L. N. Lyketsos C. G. Detroit Expert Panel on Assessment and Management of Neuropsychiatric Symptoms of Dementia (2014). Management of neuropsychiatric symptoms of dementia in clinical settings: recommendations from a multidisciplinary expert panel. J. Am. Geriatr. Soc. 62 (4), 762–769. 10.1111/jgs.12730 24635665 PMC4146407

[B64] Kales H. C. Gitlin L. N. Lyketsos C. G. (2015). Assessment and management of behavioral and psychological symptoms of dementia. BMJ 350, h369. 10.1136/bmj.h369 25731881 PMC4707529

[B65] Kaufer D. (1998). Beyond the cholinergic hypothesis: the effect of metrifonate and other cholinesterase inhibitors on neuropsychiatric symptoms in Alzheimer’s disease. Dement. Geriatr. Cogn. Disord. 9 (suppl 2), 8–14. 10.1159/000051193 9718229

[B66] Kaufer D. Cummings J. L. Christine D. (1996). Effect of tacrine on behavioral symptoms in Alzheimer’s disease: an open-label study. J. Geriatr. Psychiatry Neurol. 9 (1), 1–6. 10.1177/089198879600900101 8679057

[B67] Kaufer D. Cummings J. L. Christine D. (1998). Differential neuropsychiatric symptom responses to tacrine in Alzheimer’s disease: relationship to dementia severity. J. Neuropsychiatry Clin. Neurosci. 10 (1), 55–63. 10.1176/jnp.10.1.55 9547467

[B68] Kirven L. E. Montero E. F. (1973). Comparison of thioridazine and diazepam in the control of nonpsychotic symptoms associated with senility: double-blind study. J. Am. Geriatr. Soc. 21, 546–551. 10.1111/j.1532-5415.1973.tb01661.x 4584169

[B69] Lanctôt K. L. Chau S. A. Herrmann N. Drye L. T. Rosenberg P. B. Scherer R. W. (2014). Effect of methylphenidate on attention in apathetic AD patients in a randomized, placebo-controlled trial. Int. Psychogeriatry 26 (2), 239–246. 10.1017/S1041610213001762 PMC3927455 24169147

[B70] Lanctôt K. L. Agüera-Ortiz L. Brodaty H. Francis P. T. Geda Y. E. Ismail Z. (2017). Apathy associated with neurocognitive disorders: Recent progress and future directions. Alzheimers Dement. 13 (1), 84–100. 10.1016/j.jalz.2016.05.008 27362291

[B71] Landes A. M. Sperry S. D. Strauss M. E. Geldmacher D. S. (2001). Apathy in Alzheimer’s disease. J. Am. Geriatr. Soc. 49 (12), 1700–1707. 10.1046/j.1532-5415.2001.49282.x 11844006

[B72] Le Heron C. Apps M. A. J. Husain M. (2018). The anatomy of apathy: A neurocognitive framework for amotivated behaviour. Neuropsychologia 118 (Pt B), 54–67. 10.1016/j.neuropsychologia.2017.07.003 28689673 PMC6200857

[B73] Levy R. Dubois B. (2006). Apathy and the functional anatomy of the prefrontal cortex-basal Ganglia circuits. Cereb. Cortex 16 (7), 916–928. 10.1093/cercor/bhj043 16207933

[B74] Lopez O. L. Mackell J. A. Sun Y. Kassalow L. M. Xu Y. McRae T. (2008). Effectiveness and safety of donepezil in Hispanic patients with Alzheimer’s disease: a 12-week open-label study. J. Natl. Med. Assoc. 100 (11), 1350–1358. 10.1016/S0027-9684(15)31515-7 19024233 PMC2685180

[B75] Lovett W. C. Stokes D. K. Taylor L. B. Young M. L. Free S. M. Phelan D. G. (1987). Management of behavioral symptoms in disturbed elderly patients: comparison of trifluoperazine and haloperidol. J. Clin. Psychiatry 48, 234–236. 3294815

[B76] Lyketsos C. G. DelCampo L. Steinberg M. Miles Q. Steele C. D. Munro C. (2003). Treating depression in Alzheimer disease: efficacy and safety of sertraline therapy, and the benefits of depression reduction: the DIADS. Arch. Gen. Psychiatry 60 (7), 737–746. 10.1001/archpsyc.60.7.737 12860778

[B77] Lyketsos C. G. Steinberg M. Tschanz J. T. Norton M. C. Steffens D. C. Breitner J. C. (2000). Mental and behavioral disturbances in dementia: findings from the Cache County Study on Memory in Aging. Am. J. Psychiatry 157 (5), 708–714. 10.1176/appi.ajp.157.5.708 10784462

[B78] Lyketsos C. G. Lopez O. Jones B. Fitzpatrick A. L. Breitner J. DeKosky S. (2002). Prevalence of neuropsychiatric symptoms in dementia and mild cognitive impairment: results from the cardiovascular health study. JAMA 288 (12), 1475–1483. 10.1001/jama.288.12.1475 12243634

[B79] Lyketsos C. G. Rosenblatt A. Rabins P. (2004). Forgotten frontal lobe syndrome or ‘‘executive dysfunction syndrome’’. Psychosomatics 45 (3), 247–255. 10.1176/appi.psy.45.3.247 15123852

[B80] Lyketsos C. G. Carrillo M. C. Ryan J. M. Khachaturian A. S. Trzepacz P. Amatniek J. (2011). Neuropsychiatric symptoms in Alzheimer’ disease. Alzheimers Dement. 7 (5), 532–539. 10.1016/j.jalz.2011.05.2410 21889116 PMC3299979

[B81] Maher C. Sherrington C. Herbert R. Moseley A. Elkins M. (2003). Reliability of the PEDro scale for rating quality of randomized controlled trials. Phys. Ther. 83 (8), 713–721. 10.1093/ptj/83.8.713 12882612

[B82] Marin R. S. (1990). Differential diagnosis and classification of apathy. Am. J. Psychiatry 147 (1), 22–30. 10.1176/ajp.147.1.22 2403472

[B83] Matsuzono K. Hishikawa N. Ohta Y. Hishikawa N. Koike M. Sato K. (2015a). Combination therapy of cholinesterase inhibitor (donepezil or galantamine) plus memantine in the Okayama Memantine Study. J. Alzheimers Dis. 45, 771–780. 10.3233/JAD-143084 25624417

[B84] Matsuzono K. Yamashita T. Ohta Y. Hishikawa N. Sato K. Kono S. (2015b). Clinical benefits for older Alzheimer’s disease patients: Okayama Late Dementia Study (OLDS). J. Alzheimers Dis. 46 (3), 687–693. 10.3233/JAD-150175 26402513

[B85] Matthews H. P. Korbey J. Wilkinson D. G. Rowden J. (2000). Donepezil in Alzheimer’s disease: eighteen month results from Southampton Memory Clinic. Int. J. Geriatr. Psychiatry 15 (8), 713–720. 10.1002/1099-1166(200008)15:8<713::AID-GPS187>3.0.CO;2-I 10960883

[B86] Medicine OCfE-b (2009). Levels of evidence and grades of recommendation. Oxford Centre for Evidence-Based Medicine. University of Oxford 2009 https://www.cebm.net/2009/06/oxford-centre-evidence-based-medicine-levels-evidence-march-2009/ Accessed April 27, 2019.

[B87] Mega M. S. Cummings J. L. Fiorello T. Gornbein J. (1996). The spectrum of behavioral changes in Alzheimer’s disease. Neurology 46 (1), 130–135. 10.1212/WNL.46.1.130 8559361

[B88] Mega M. S. Masterman D. M. O’Connor S. M. Barclay T. R. Cummings J. L. (1999). The spectrum of behavioral responses to cholinesterase inhibitor therapy in Alzheimer disease. Arch. Neurol. 56 (11), 1388–1393. 10.1001/archneur.56.11.1388 10555660

[B89] Mega M. S. Cummings J. L. Salloway S. Malloy P. (1997). The limbic system: an anatomic, phylogenetic, and clinical perspective. J. Neuropsychiatry Clin. Neurosci. 9 (3), 315–330. 10.1212/WNL.46.1.130 9276837

[B90] Monsch A. U. Giannakopoulos P. Study Group, GAL-SUI (2004). Effects of galantamine on behavioural and psychological disturbances and caregiver burden in patients with Alzheimer’s disease. Curr. Med. Res. Opin. 20 (6), 931–938. 10.1185/030079904125003890 15200752

[B91] Moretti R. Torre P. Antonello R. M. Cazzato G. Bava A. (2003). Gabapentin for the treatment of behavioural alterations in dementia: preliminary 15-month investigation. Drugs Aging 20, 1035–1040. 10.2165/00002512-200320140-00003 14651443

[B92] Morris J. C. Cyrus P. A. Orazem J. Mas J. Bieber F. Ruzicka B. B. (1998). Metrifonate benefits cognitive, behavioral, and global function in patients with Alzheimer’s disease. Neurology 50 (5), 1222–1230. 10.1212/WNL.50.5.1222 9595967

[B93] Mulin E. Leone E. Dujardin K. Delliaux M. Leentjens A. Nobili F. (2011). Diagnostic criteria for apathy in clinical practice. Int. J. Geriatr. Psychiatry 26 (2), 158–165. 10.1002/gps.2508 20690145

[B94] Negron A. E. Reichman W. E. (2000). Risperidone in the treatment of patients with Alzheimer’s disease with negative symptoms. Int. Psychogeriatry 12 (4), 527–536. 10.1017/S1041610200006633 11263718

[B95] Niu Y. X. Tan J. P. Guan J. Q. Zhang Z. Q. Wang L. N. (2010). Cognitive stimulation therapy in the treatment of neuropsychiatric symptoms in Alzheimer’s disease: a randomized controlled trial. Clin. Rehabil. 24 (12), 1102–1111. 10.1177/0269215510376004 20713437

[B96] Nobis L. Husain M. (2018). Apathy in Alzheimer’s disease. Curr. Opin. Behav. Sci. 22, 7–13. 10.1016/j.cobeha.2017.12.007 30123816 PMC6095925

[B97] Nyth A. L. Gottfries C. G. Lyby K. Smedegaard-Andersen L. Gylding-Sabroe J. Kristensen M. (1992). A controlled multicenter clinical study of citalopram and placebo in elderly depressed patients with and without concomitant dementia. Acta Psychiatry Scand. 86 (2), 138–145. 10.1111/j.1600-0447.1992.tb03242.x 1529737

[B98] Oberholzer A. F. Hendriksen C. Monsch A. U. Heierli B. Stahelin H. B. (1992). Safety and effectiveness of low-dose clozapine in psychogeriatric patients: a preliminary study. Int. Psychogeriatry 1992, 4 (2), 187–195. 10.1017/S1041610292001017 1477306

[B99] Onor M. L. Saina M. Trevisiol M. Cristante T. Aguglia E. (2007). Clinical experience with risperidone in the treatment of behavioural and psychological symptoms of dementia. Prog. Neuropsychopharmacol. Biol. Psychiatry 31 (1), 205–209. 10.1016/j.pnpbp.2006.09.001 17020789

[B100] Onyike C. U. Sheppard J. M. E. Tschanz J. T. Norton M. C. Green R. C. Steinberg M. (2007). Epidemiology of apathy in older adults: the Cache County Study. Am. J. Ger. Psychiatry 15 (5), 365–375. 10.1097/01.JGP.0000235689.42910.0d 17463187

[B101] Padala P. R. Burke W. J. Shostrom V. K. Bhatia S. C. Wengel S. P. Potter J. F. (2010). Methylphenidate for apathy and functional status in dementia of the Alzheimer type. Am. J. Geriatr. Psychiatry 18 (4), 371–374. 10.1097/JGP.0b013e3181cabcf6 20220576

[B102] Padala P. R. Padala K. P. Lensing S. Y. Ramirez D. Monga V. Bopp M. M. (2017). Methylphenidate for apathy in community-dwelling older veterans with mild Alzheimer’s disease: a double-blind, randomized, placebo-controlled trial. Am. J. Psychiatry 175 (2), 159–168. 10.1176/appi.ajp.2017.17030316 28945120

[B103] Paleacu D. Mazeh D. Mirecki I. Even M. Barak Y. (2002). Donepezil for the treatment of behavioral symptoms in patients with Alzheimer’s disease. Clin. Neuropharmacol. 25 (6), 313–317. 10.1097/00002826-200211000-00007 12469005

[B104] Pantev M. Ritter R. Gortelmeyer R. (1993). Clinical and behavioural evaluation in long-term care patients with mild to moderate dementia under memantine treatment. Zeitschrt für Gerontopsychologie Und-Psychiatrie 6 (2), S103–S117.

[B105] Pantoni L. Carosi M. Amigoni S. Mascalchi M. Inzitari D. (1996). A preliminary open trial with nimodipine in patients with cognitive impairment and leukoaraiosis. Clin. Neuropharmacol. 19, 497–506. 10.1097/00002826-199619060-00003 8937788

[B106] Peskind E. R. Potkin S. G. Pomara N. Ott B. R. Graham S. M. Olin J. T. (2006). Memantine treatment in mild to moderate Alzheimer disease: a 24-week randomized, controlled trial. Am. J. Geriatr. Psychiatry 14 (8), 704–715. 10.1097/01.JGP.0000224350.82719.83 16861375

[B107] Petrie W. M. Ban T. A. Berney S. Fujimori M. Guy W. Ragheb M. (1982). Loxapine in psychogeriatrics: a placebo- and standard-controlled clinical investigation. J. Clin. Psychopharmacol. 2, 122–126. 10.1097/00004714-198204000-00008 7042770

[B108] Politis A. M. Mayer L. S. Passa M. Maillis A. Lyketsos C. G. (2004). Validity and reliability of the newly translated Hellenic Neuropsychiatric Inventory (H-NPI) applied to Greek outpatients with Alzheimer’s disease: a study of disturbing behaviors among referrals to a memory clinic. Int. J. Geriatr. Psychiatry 19 (3), 203–208. 10.1002/gps.1045 15027034

[B109] Pollock B. G. Mulsant B. H. Rosen J. Sweet R. A. Mazumdar S. Bharucha A. (2002). Comparison of citalopram, perphenazine, and placebo for the acute treatment of psychosis and behavioral disturbances in hospitalized, demented patients. Am. J. Psychiatry 159 (3), 460–465. 10.1176/appi.ajp.159.3.460 11870012

[B110] Porsteinsson A. P. Grossberg G. T. Mintzer J. Olin J. T. (2008). Memantine MEM-MD-12 Study Group. Memantine treatment in patients with mild to moderate Alzheimer’s disease already receiving a cholinesterase inhibitor: a randomized, doubleblind, placebo-controlled trial. Curr. Alzheimer Res. 5 (1), 83–89. 10.2174/156720508783884576 18288936

[B111] Porsteinsson A. P. Drye L. T. Pollock B. G. Devanand D. P. Frangakis C. Ismail Z. (2014). Effect of citalopram on agitation in Alzheimer’s disease—the CitAD randomized controlled trial. JAMA 311 (7), 682–691. 10.1001/jama.2014.93 24549548 PMC4086818

[B112] Radakovic R. Harley C. Abrahams S. Starr J. M. (2015). A systematic review of the validity and reliability of apathy scales in neurodegenerative conditions. Int. Psychogeriatry 27 (6), 903–923. 10.1017/S1041610214002221 25355282

[B113] Raskind M. A. Cyrus P. A. Ruzicka B. B. Gulanski B. I. (1999). The effects of metrifonate on the cognitive, behavioral, and functional performance of Alzheimer’s disease patients. Metrifonate Study Group. J. Clin. Psychiatry 60 (5), 318–325. 10.4088/JCP.v60n0510 10362441

[B114] Rea R. Carotenuto A. Traini E. Fasanaro A. M. Manzo V. Amenta F. (2015). Apathy treatment in Alzheimer’s disease: interim results of the ASCOMALVA trial. J. Alzheimers Dis. 48, 377–383. 10.3233/JAD-141983 26402001

[B115] Reisberg B. Doody R. Stoffler A. Schmitt F. Ferris S. Möbius H. J. (2003). Memantine in moderate-to severe Alzheimer’s disease. New Engl. J. Med. 348 (14), 1333–1341. 10.1056/NEJMoa013128 12672860

[B116] Robert P. Lanctôt K. L. Agüera-Ortiz L. Aalten P. Bremond F. Defrancesco M. (2018). Is it time to revise the diagnostic criteria for apathy in brain disorders? The 2018 international consensus group. Eur. Psychiatry 54, 71–76. 10.1016/j.eurpsy.2018.07.008 30125783

[B117] Rockwood K. Mintzer J. Truyen L. Wessel T. Wilkinson D. (2001). Effects of a flexible galantamine dose in Alzheimer’s disease: a randomised, controlled trial. J. Neurol. Neurosurg. Psychiatry 71 (5), 589–595. 10.1136/jnnp.71.5.589 11606667 PMC1737604

[B118] Rockwood K. Black S. Bedard M. A. Tran T. Lussier I. Study Investigators T. O. P. S. (2007). Specific symptomatic changes following donepezil treatment of Alzheimer’s disease: a multicentre, primary care, open label study. Int. J. Geriatr. Psychiatry 22 (4), 312–319. 10.1002/gps.1675 17006874

[B119] Rosenberg P. B. Lanctôt K. L. Drye L. T. Herrmann N. Scherer R. W. Bachman D. L. (2013). Safety and efficacy of methylphenidate for apathy in Alzheimer’s disease: a randomized, placebo-controlled trial. J. Clin. Psychiatry 74 (8), 810–816. 10.4088/JCP.12m08099 24021498 PMC3902018

[B120] Rosenberg P. B. Lyketsos C. G. (2008). Mild cognitive impairment: searching for the prodrome of Alzheimer’s disease. World Psychiatry 7 (2), 72–78. 10.1002/j.2051-5545.2008.tb00159.x 18563204 PMC2430721

[B121] Ruthirakuhan M. T. Herrmann N. Abraham E. H. Chan S. Lanctôt K. L. (2018). Pharmacological interventions for apathy in Alzheimer’s disease. Cochrane Database Syst. Rev. 5, Cd012197. 10.1002/14651858.CD012197.pub2 29727467 PMC6494556

[B122] Schmidt R. Baumhackl U. Berek K. Brücke T. Kapeller P. Lechner A. (2010). Memantine for treatment of behavioural disturbances and psychotic symptoms in moderate to moderately severe Alzheimer dementia: a naturalistic study in outpatient services in Austria [in German]. Neuropsychiatry 24 (2), 125–131. 20605008

[B123] Schulz K. F. Chalmers I. Hayes R. J. Altman D. G. (1995). Empirical evidence of bias. Dimensions of methodological quality associated with estimates of treatment effects in controlled trials. JAMA 273 (5), 408–412. 10.1001/jama.1995.03520290060030 7823387

[B124] Scripnikov A. Khomenko A. Napryeyenko O. GINDEM-NP Study Group (2007). Effects of Ginkgo biloba extract EGb 761 on neuropsychiatric symptoms of dementia: findings from a randomized controlled trial. Wien. Med. Wochenschr. 157 (13–14), 295–300. 10.1007/s10354-007-0427-5 17704975

[B125] Seltzer B. Zolnouni P. Nunez M. Goldman R. Kumar D. Ieni J. (2004). Efficacy of donepezil in early-stage Alzheimer disease: a randomized placebo-controlled trial. Arch. Neurol. 61 (12), 1852–1856. 10.1001/archneur.61.12.1852 15596605

[B126] Sepehry A. A. Sarai M. Hsiung G. R. (2017). Pharmacological therapy for apathy in Alzheimer’s disease: a systematic review and meta-analysis. Can. J. Neurol. Sci. 44, 267–275. 10.1017/cjn.2016.426 28148339

[B127] Sherman C. Liu C. S. Herrmann N. Lanctôt K. L. (2018). Prevalence, neurobiology, and treatments for apathy in prodromal dementia. Int. Psychogeriatry 30 (2), 177–184. 10.1017/S1041610217000527 28416030

[B128] Siddique H. Hynan L. S. Weiner M. F. (2009). Effect of a serotonin reuptake inhibitor on irritability, apathy and psychotic symptoms in patients with Alzheimer’s disease. J. Clin. Psychiatry 70 (6), 915–918. 10.4088/JCP.08m04828 19422762 PMC3236068

[B129] Sival R. Haffmans P. M. Jansen P. A. Duursma S. A. Eikelenboom P. (2002). Sodium valproate in the treatment of aggressive behavior in patients with dementia—a randomized placebo controlled clinical trial. Int. J. Geriatr. Psychiatry 17, 579–585. 10.1002/gps.653 12112183

[B130] Sockeel P. Dujardin K. Devos D. Denève C. Destée A. Defebvre L. (2006). The Lille apathy rating scale (LARS), a new instrument for detecting and quantifying apathy: validation in Parkinson’s disease. J. Neurol. Neurosurg. Psychiatry 77, 579–584. 10.1136/jnnp.2005.075929 16614016 PMC2117430

[B131] Staekenborg S. S. Su T. van Straaten E. C. Lane R. Scheltens P. Barkhof F. (2010). Barkhof, FBehavioral and psychological symptoms in vascular dementia; differences between small- and large-vessel disease. J. Neurol. Neurosurg. Psychiatry 81, 547–552. 10.1136/jnnp.2009.187500 19965852

[B132] Starkstein S. E. Petracca G. Chemerinski E. Kremer J. (2001). Syndromic validity of apathy in Alzheimer’s disease. Am. J. Psychiatry 158 (6), 872–877. 10.1176/appi.ajp.158.6.872 11384893

[B133] Tanaka M. Namiki C. Thuy D. H. Yoshida H. Kawasaki K. Hashikawa K. (2004). Prediction of psychiatric response to donepezil in patients with mild to moderate Alzheimer’s disease. J. Neurol. Sci. 225 (1–2), 135–141. 10.1016/j.jns.2004.07.009 15465097

[B134] Tariot P. N. Cohen R. M. Sunderland T. Newhouse P. A. Yount D. Mellow A. M. (1987). L-deprenyl in Alzheimer’s disease. Preliminary evidence for behavioral change with monoamine oxidase B inhibition. Arch. Gen. Psychiatry 44 (5), 427–433. 10.1001/archpsyc.1987.01800170041007 3107514

[B135] Tariot P. N. Cummings J. L. Katz I. R. Mintzer J. Perdomo C. A. Schwam E. M. (2001). A randomized, double blind, placebo-controlled study of the efficacy and safety of donepezil in patients with Alzheimer’s disease in the nursing home setting. J. Am. Geriatr. Soc. 49 (12), 1590–1599. 10.1111/j.1532-5415.2001.49266.x 11843990

[B136] Tariot P. N. Solomon P. R. Morris J. C. Kershaw P. Lilienfeld S. Ding C. (2000). A 5-month, randomized, placebo-controlled trial of galantamine in AD. The Galantamine USA-10 Study Group. Neurology 54 (12), 2269–2276. 10.1212/WNL.54.12.2269 10881251

[B137] Tariot P. N. Farlow M. R. Grossberg G. T. Graham S. M. McDonald S. Gergel I. (2004). Memantine treatment in patients with moderate to severe Alzheimer disease already receiving donepezil: a randomized controlled trial. JAMA 291 (3), 317–324. 10.1001/jama.291.3.317 14734594

[B138] Theleritis C. Politis A. Siarkos K. Lyketsos C. G. (2014). A review of neuroimaging findings of apathy in Alzheimer’s disease. Int. Psychogeriatry 26 (2), 195–207. 10.1017/S1041610213001725 PMC4086515 24135083

[B139] Theleritis C. Siarkos K. Politis A. A. Katirtzoglou E. Politis A. (2018). A systematic review of non-pharmacological treatments for apathy in dementia. Int. J. Geriatr. Psychiatry 33 (2), e177–e192. 10.1002/gps.4783 28960446

[B140] Theleritis C. Siarkos K. Katirtzoglou E. Politis A. (2017). Pharmacological and non-pharmacological treatment for apathy in Alzheimer’s disease. A systematic review across modalities. J. Geriatr. Psychiatry Neurol. 30 (1), 26–49. 10.1177/0891988716678684 28248559

[B141] Trifiro G. Spina E. Gambassi G. (2009). Use of antipsychotics in elderly patients with dementia: do atypical and conventional agents have a similar safety profile? Pharmacol. Res. 59 (1), 1–12. 10.1016/j.phrs.2008.09.017 18977443

[B142] Trifiro G. Sultana J. Spina E. (2014). Are the safety profiles of antipsychotic drugs used in dementia the same? An updated review of observational studies. Drug Saf. 37 (7), 501–520. 10.1007/s40264-014-0170-y 24859163

[B143] Trinh N.H. Hobbin J. Mohanty S. Yaffe K. (2003). Efficacy of cholinesterase inhibitors in the treatment of neuropsychiatric symptoms and functional impairment in Alzheimer disease: a metaanalysis. J.A.M.A. 289 (2), 210–216. 10.1001/jama.289.2.210 12517232

[B144] Waldemar G. Gauthier S. Jones R. Wilkinson D. Cummings J. Lopez O. (2011). Effect of donepezil on emergence of apathy in mild to moderate Alzheimer’s disease. Int. J. Geriatr. Psychiatry 26 (2), 150–157. 10.1002/gps.2507 20597141

[B145] Weiner M. F. Martin-Cook K. Foster B. M. Saine K. Fontaine C. S. Svetlik D. A. (2000). Effects of donepezil on emotional/behavioral symptoms in Alzheimer’s disease patients. J. Clin. Psychiatry 61 (7), 487–492. 10.4088/JCP.v61n0705 10937606

[B146] Winblad B. Engedal K. Soininen H. Verhey F. Waldemar G. Wimo A. (2001). A 1-year, randomized, placebo controlled study of donepezil in patients with mild to moderate AD. Neurology 57 (3), 489–495. 10.1212/WNL.57.3.489 11502918

[B147] Winblad B. Poritis N. (1999). Memantine in severe dementia: results of the 9M-Best Study (Benefit and efficacy in severely demented patients during treatment with memantine). Int. J. Geriatr. Psychiatry 14 (2), 135–146. 10.1002/(SICI)1099-1166(199902)14:2<135::AID-GPS906>3.0.CO;2-0 10885864

[B148] Wongpakaran N. van Reekum R. Wongpakaran T. Clarke D. (2007). Selective serotonin reuptake inhibitor use associates with apathy among depressed elderly: a case-control study. Ann. Gen. Psychiatry 6, 7. 10.1186/1744-859X-6-7 17313684 PMC1820592

[B149] van Dyck C. H. Tariot P. N. Meyers B. Malca R. E. Memantine MEM-MD-01 Study Group (2007). A 24-week randomized, controlled trial of memantine in patients with moderate-to-severe Alzheimer disease. Alzheimer Dis. Assoc. Disord. 21 (2), 136–143. 10.1097/WAD.0b013e318065c495 17545739

[B150] Zhou T. Wang J. Xin C. Kong L. Wang C. (2019). Effect of memantine combined with citalopram on cognition of BPSD and moderate Alzheimer’s disease: a clinical trial. Exp. Ther. Med. 17 (3), 1625–1630. 10.3892/etm.2018.7124s 30783429 PMC6364245

